# Inelastic phonon transport across atomically sharp metal/semiconductor interfaces

**DOI:** 10.1038/s41467-022-32600-w

**Published:** 2022-08-20

**Authors:** Qinshu Li, Fang Liu, Song Hu, Houfu Song, Susu Yang, Hailing Jiang, Tao Wang, Yee Kan Koh, Changying Zhao, Feiyu Kang, Junqiao Wu, Xiaokun Gu, Bo Sun, Xinqiang Wang

**Affiliations:** 1 Tsinghua-Berkeley Shenzhen Institute, Tsinghua University, Shenzhen, 518055 China; 2 State Key Laboratory for Mesoscopic Physics and Frontiers Science Center for Nano-optoelectronics, School of Physics, Peking University, Beijing, 100871 China; 3 Collaborative Innovation Center of Quantum Matter, Beijing, 100871 China; 4 Institute of Engineering Thermophysics, School of Mechanical Engineering, Shanghai Jiao Tong University, Shanghai, 200240 China; 5 Electron Microscopy Laboratory, School of Physics, Peking University, Beijing, 100871 China; 6 Department of Mechanical Engineering and Center of Advanced 2D Materials, National University of Singapore, Singapore, 117576 Singapore; 7 Tsinghua Shenzhen International Graduate School and Guangdong Provincial Key Laboratory of Thermal Management Engineering & Materials, Shenzhen, 518055 China; 8 Department of Materials Science and Engineering, University of California, Berkeley, CA 94720 USA; 9 Materials Sciences Division, Lawrence Berkeley National Laboratory, Berkeley, CA 94720 USA

**Keywords:** Semiconductors, Electronic devices

## Abstract

Understanding thermal transport across metal/semiconductor interfaces is crucial for the heat dissipation of electronics. The dominant heat carriers in non-metals, phonons, are thought to transport elastically across most interfaces, except for a few extreme cases where the two materials that formed the interface are highly dissimilar with a large difference in Debye temperature. In this work, we show that even for two materials with similar Debye temperatures (Al/Si, Al/GaN), a substantial portion of phonons will transport inelastically across their interfaces at high temperatures, significantly enhancing interface thermal conductance. Moreover, we find that interface sharpness strongly affects phonon transport process. For atomically sharp interfaces, phonons are allowed to transport inelastically and interface thermal conductance linearly increases at high temperatures. With a diffuse interface, inelastic phonon transport diminishes. Our results provide new insights on phonon transport across interfaces and open up opportunities for engineering interface thermal conductance specifically for materials of relevance to microelectronics.

## Abstract (web summary)

Phonons are thought to transport elastically across most interfaces. Here, the authors show that a substantial portion of phonons transport inelastically, adding another heat conduction channel and enhancing thermal conductance across interfaces.

## Introduction

In modern electronics, thermal resistance of interfaces (reciprocal of thermal conductance) is the main limiting factor for heat dissipation, especially for power electronics and high-energy density applications^1–5^. The scattering of heat carriers, predominately phonons, leads to interface thermal resistance. Over the past several decades, there were extensive studies on thermal conductance across metal/semiconductor interfaces, both experimentally and theoretically^6–12^. However, the rare agreement between experiments and calculations, as well as the scattered experimental results even for the same interface, warrant study for a much better understanding of thermal transport across interfaces^6,13–15^.

Theories have been developed to explain interface thermal conductance since the 1950s, such as the widely used acoustic mismatch model (AMM) and diffuse mismatch model (DMM)^16,17^. AMM is based on the assumption that phonon is reflected or transmitted specularly, while DMM assumes that phonon scattering is completely diffusive at the interface. Both DMM and AMM assume phonon transport across the interface is elastic, which means the transmitted/reflected phonon has the same frequency as the incident phonon. The elastic transport assumption predicts that the interface thermal conductance will reach a plateau at temperatures higher than the lower Debye temperature (*T*_D_) of the two materials that formed the interface, when all phonons in this side have been excited. In recent years, advanced calculation methods such as molecular dynamics (MD) and atomistic Green function (AGF) have been used to study phonon transport process across interfaces^6–8,10,11,18^. The inelastic phonon transport process has been predicted to exist in interfaces between very dissimilar materials, where the transmitted phonons do not have the same frequency as the incident phonons^7,11,19^, and anharmonicity was found to be of fundamental importance for the inelastic phonon transport across interfaces. Despite these advances, there are still controversies related to under what conditions the inelastic process will occur in the first place. For example, Landry and McGaughey predicted that inelastic phonon transport becomes dominant when the temperature is higher than ~500 K, whereas Feng and Ruan computed that inelastic phonon transport contributes more than 50% to the total thermal conductance for Si/Ge interfaces even at room temperature^7,11^.

Most experimental results show that phonon transport across metal/semiconductor interface is an elastic process, as interface thermal conductance saturates at high temperatures for most interfaces under study^6,15,20^. There are only a few exceptions to this trend^12,13,21^, and all of these suggest a large Debye temperature difference across interfaces leads to the observation of non-saturated thermal conductance at high temperatures. The most notable one is the highly dissimilar Bi/diamond interface (the *T*_D_ ratio of diamond and Bi is ~19), where Lyeo and Cahill observed a linear increase of thermal conductance with temperature^12^. This is attributed to the temperature-dependent inelastic phonon transport, which adds an additional thermal transport channel across interfaces^12,16^. However, it is still an open question how large a Debye temperature difference is required for inelastic phonon transport to occur, which needs to be examined in detail.

The lack of high-quality interfaces limited the experimental study of phonon transport across interfaces. Extrinsic phonon scattering centers, such as atomic intermixing, interface roughness and contamination, would easily scatter phonons and bury the phonon’s intrinsic elastic and inelastic transport processes across interfaces^15^. Epitaxial metal/semiconductor interfaces are usually used to study the intrinsic interface phonon transport due to their importance and high quality^6,15,22,23^. However, previous studies on epitaxial interfaces used typically lacked atomic-level structural details^12,15,21^ with interface roughness and atomic intermixing often ignored, thus only lead to qualitative analysis and limit our understanding of intrinsic phonon transport across interfaces.

Here, we report the observation of inelastic phonon transport across metal/semiconductor interfaces, with a clear crossover from elastic-dominated to inelastic-dominated phonon transport following the rise of temperature. Our results show that, even in an interface formed with highly similar materials with a Debye temperature ratio <1.5, inelastic phonon transport still exists and significantly enhances thermal conductance at high temperatures, suggesting that inelastic phonon transport is universal across interfaces even in acoustically similar materials. We also observed that inelastic phonon transport tends to dominate the process when the interface is atomically sharp. Our MD simulations confirmed that the interface sharpness is crucial for inelastic phonon transport, as phonon non-equilibrium is more likely to happen near the sharp interface.

## Results

We built high-quality metal/semiconductor interfaces by epitaxial growth of Al(111) on Si(111) and GaN(0001) using molecular beam epitaxy (MBE), see Methods and Supplementary Information Note I. Before the growth of Al/Si interface, a Si wafer was cleaned by hydrofluoric acid and then heated in a vacuum at 900 °C to ensure the surface was free of oxide layer and adsorbates. To fabricate an Al/Si interface with controlled interface quality, Al growth was proceeded at different temperatures, 100 °C (denoted as Sample 1) and 300 °C (denoted as Sample 2), knowing that 100 °C is the optimum temperature for Al deposition in our prior work^24^. For Al/GaN interface, GaN thin film was grown on a sapphire substrate at 800 °C in the MBE chamber first, and then the temperature was ramped down to 150 °C to grow the Al layer. For comparison, an Al/SiO_2_/Si sample was also prepared by e-beam evaporation of Al on Si substrate in presence of native oxide.

**Fig. 1:**
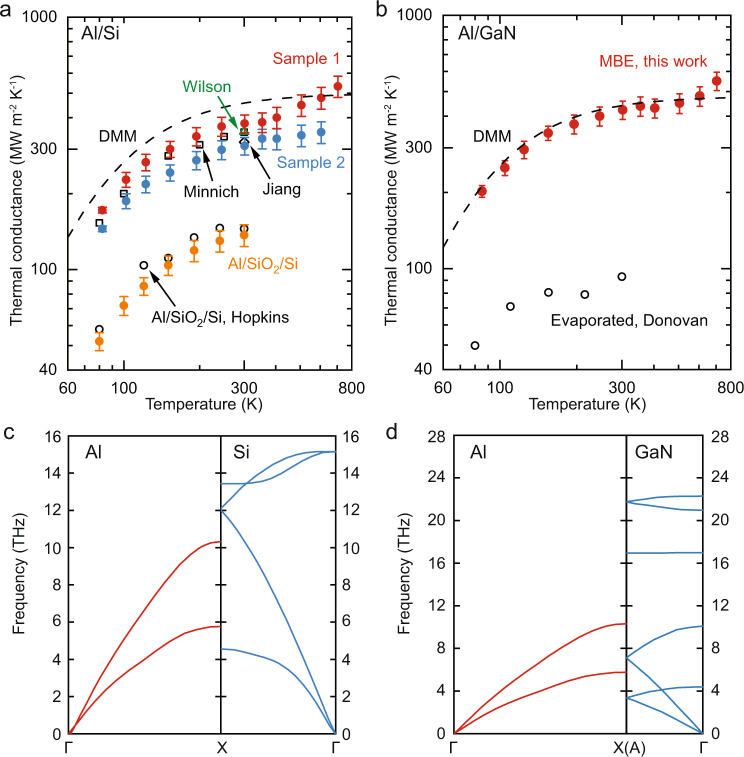
Thermal conductance of Al/Si and Al/GaN interfaces. **a** Thermal conductance of Al/Si Sample 1 (red spheres) and Sample 2 (blue spheres). Black dashed line is interface thermal conductance calculated by DMM. For comparison, we show previously measured Al/Si thermal conductance in open squares by Minnich^39^,  triangle by Wilson^40^, and diamond by Jiang^41^. Yellow solid spheres are measured thermal conductance of Al/Si with a native oxide layer, compared with the results by Hopkins, shown in open circles^14^. **b** Thermal conductance of Al/GaN interface (red spheres). For comparison, the calculated thermal conductance using DMM (black dashed line) is plotted. Previous measurement results by Donovan^42^ are shown in open circles, the Al film of which was deposited by e-beam evaporation. Phonon dispersion relations of Al/Si (**c**) and Al/GaN (**d**) are calculated from first-principles. The calculation of error bars is detailed in Supplementary Information Note VIII.

We measured the thermal conductance of Al/Si and Al/GaN interfaces over a wide range of temperatures (80–700 K) by time-domain thermoreflectance (TDTR)^25,26^. The raw TDTR data, data analysis, and uncertainty estimation can be found in Methods and Supplementary Information Note V-VIII. The measured thermal conductance G of Al/Si and Al/GaN interfaces as a function of temperature are plotted in Fig. 1a and Fig. 1b. At room temperature, Al/Si Sample 1 and Al/GaN show a record high thermal conductance of 379 and 423 MW m^−2^ K^−1^, respectively. For both Al/Si Sample 1 and Al/GaN, our results can be clearly divided into two regimes. At temperatures lower than the Debye temperature of Al (*T*_D_ = 428 K), the thermal conductance of Al/Si Sample 1 and Al/GaN gradually saturate with the increase of temperature, which has the same trend as the previous measured thermal conductance of Al/Si and Al/GaN interfaces. However, when the temperature approaches 400 K (close to the Debye temperature of Al) and beyond, both Al/Si Sample 1 and Al/GaN show a linear increase in thermal conductance with temperature instead of reaching a plateau. For Al/Si Sample 2, it has a thermal conductance of 309 MW m^−2^ K^−1^ at room temperature. Throughout the low temperatures (*T* < *T*_D_), the thermal conductance of Al/Si Sample 2 is ~10% lower than that of Al/Si Sample 1. However, unlike Al/Si Sample 1 and Al/GaN, Al/Si Sample 2 shows a saturated thermal conductance when *T* > *T*_D_.

For the metal/semiconductor interface, there are four thermal transport interactions, which are the phonon–phonon transport across interface including both elastic and inelastic phonon transport, as well as the electron–phonon coupling in the metal and across interfaces. The effect of electron–phonon coupling across interfaces is negligible^13^. The electron–phonon coupling in Al adds an additional thermal resistance in series with the phonon–phonon interactions^27^, which is driven by the thermal non-equilibrium between electrons and phonons near the interface. To calculate the phonon–phonon transport induced interface thermal conductance alone, we followed Majumdar and Reddy’s treatment of electron–phonon coupling to use ${{{{{\rm{G}}}}}}=\frac{{{{{{{\rm{G}}}}}}}_{{{{{{\rm{ep}}}}}}}{{{{{{\rm{G}}}}}}}_{{{{{{\rm{pp}}}}}}}}{{{{{{{\rm{G}}}}}}}_{{{{{{\rm{ep}}}}}}}+{{{{{{\rm{G}}}}}}}_{{{{{{\rm{pp}}}}}}}}$, where G is the total thermal conductance, G_ep_ is electron–phonon coupling and G_pp_ is phonon–phonon transport induced thermal conductance^27^. The electron–phonon coupling induces the conductance ${{{{{{\rm{G}}}}}}}_{{{{{{\rm{ep}}}}}}}=\sqrt{{{{{{\rm{g}}}}}}{\Lambda }_{{{{{{\rm{p}}}}}}}}$, where g is the electron cooling rate and Λ_p_ is the lattice thermal conductivity of Al. We used experimentally determined g^28^ and first-principles calculated Λ_p_^29^, which have been reported previously and are well accepted, to determine G_ep_. The calculated G_pp_ for Al/Si Sample 1, Sample 2, and Al/GaN are shown in Supplementary Fig. 10. It shows that G_pp_ of Al/Si Sample 1 and Al/GaN interfaces has a stronger temperature dependence than G, while G_pp_ of Al/Si Sample 2 changes slightly with the temperature at high temperatures. Besides, the electron–phonon coupling reduces the measured G below that prediction from phonon–phonon interactions alone.

**Fig. 2:**
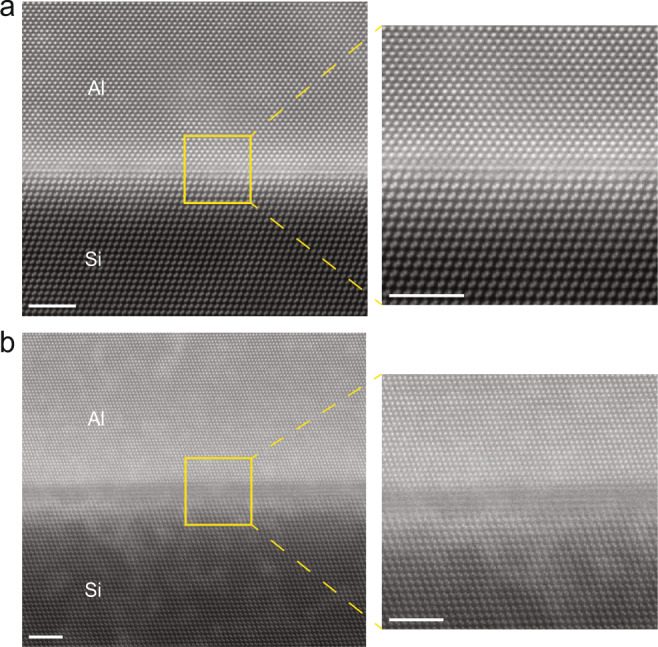
Interface structure of Al/Si Sample 1 and Sample 2. Cross-sectional TEM image of Al(111)/Si(111) Sample 1 (**a**) and Sample 2 (**b**). Scale bars are 2.5 nm.

To study the relationship between interface quality and thermal conductance and understand the difference between the two Al/Si interfaces, high-angle annular dark-field scanning transmission electron microscopy (HAADF-STEM) was used to study the cross-sectional interface structure, as shown in Fig. 2. The STEM images clearly shows that Al/Si Sample 1 has a sharp interface, while Sample 2 has a diffuse interface. The interface structure of Al/Si Sample 1 is shown in Fig. 2a, showing the interface between Al and Si is atomically sharp, with only 1–2 distorted layers of interface atoms observed. The cross-sectional HAADF-STEM image of Al/Si Sample 2 is shown in Fig. 2b and Supplementary Fig. 7. Unlike the Al/Si Sample 1, it shows a diffuse interface with an interdiffusion region of 1.38 nm and 1.2 nm for two randomly chosen spots, which roughly equals the thickness of 3–5 atomic layers of Si. Further structure analysis in Supplementary Fig. 9 shows that the diffuse interface of Sample 2 results from the intermixing of Al and Si atoms. Both interfaces of Sample 1 and Sample 2 are quite homogenous, as seen in Supplementary Figs. 3 and 7. We have monitored that there is no trace of oxygen residue at the interface (see Supplementary Fig. 1a). The strain analysis shows that the strain localizes at the nearest layers adjacent to the sharp interface, as in Supplementary Fig. 5. Though the as-grown Al films are of high quality, there are still lattice imperfections. The main imperfections in the Al layer are domains, which are micron sized and much larger than the phonon or electron mean free path in Al, thus have little effect on thermal transport.

When the samples are heated up, interdiffusion across the interface will inevitably occur. The diffused Al or Si atoms create some disorder in the other side. And according to some previous calculations, such disorder will facile phonon transport and increase interface thermal conductance^30–32^. To test this, we fabricated sharp Al/Si interfaces with both intrinsic Si and doped Si, and we found that the two interfaces have nearly identical thermal conductance (see Supplementary Fig. 11). We infer that a small portion of foreign atoms will not affect interface thermal conductance, thus the increase of thermal conductance with temperature is intrinsic.

**Fig. 3:**
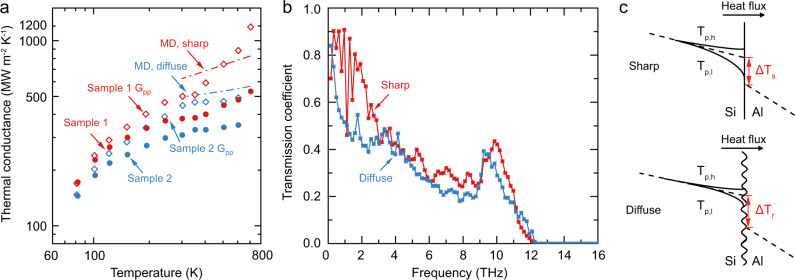
Phonon transport behavior across Al/Si interface computed by molecular dynamics. **a** Calculated thermal conductance of sharp (red dashed line) and diffuse (blue dashed line) Al/Si interfaces. **b** Phonon transmission coefficient for sharp (red) and diffuse (blue) interfaces. **c** Schematic of temperature distributions near the sharp and diffuse interfaces. Here *T*_p,h_ and *T*_p,l_ represent temperatures of high- and low-energy phonons. Δ*T*_s_ and Δ*T*_r_ are temperatures drop across sharp and diffuse interfaces.

To understand the phonon transport mechanism, we used non-equilibrium molecular dynamics (NEMD) to compute the interface thermal conductance of both sharp and diffuse Al/Si interfaces (see Methods). The Al/Si structure along the (111) orientation was established with sharp and diffuse interfaces, as shown in Supplementary Figs. 1a and 1b. The sharp interface is composed of Si(111) 3 × 3 unit cell and Al(111) 4 × 4 unit cell. Before calculating the thermal interface conductance, the structure was fully relaxed to reduce the stress at the interface. A diffuse interface was attained by locally melting the interface at 3000 K and quenched to 300 K in the MD simulation, and the thickness of the diffuse interface is about 1.3 nm. The simulation results of the sharp interface at 500 K with a heat bath temperature difference of 60 K are shown in Supplementary Information Note IX, and the thermal conductance was calculated as 738 MW m^−2^ K^−1^. The thermal conductance predicted by MD at high temperature is shown in Fig. 3a. The interface thermal conductance at the diffuse interface is lower than the value of the sharp interface, which is consistent with the experiment results. As temperature rises, the increasing slope of thermal conductance as a function of temperature at the sharp interface is much higher than that of the diffuse interface, demonstrating that the surface sharpness is crucial for the observation of inelastic phonon scattering, which occurs across the atomically sharp interfaces. Considering that MD simulation does not make assumptions on the phonon scattering mechanisms, the temperature dependence of thermal conductance at high temperatures is possibly universal, indicating that inelastic phonon scattering is always expected at high-quality interfaces.

To further explain the observed distinct temperature dependence of the thermal conductance, we calculated the spectral phonon transmissivity using atomistic Green’s function, as shown in Fig. 3b. For the sharp interface, the transmissivity of low-energy phonons (here we define phonons lower than 4 THz as low-energy phonons) is higher than the high-energy phonons. This will result in a relatively large temperature difference between these phonons, as sketched in Fig. 3c. Such a thermal non-equilibrium between high-energy phonons and low-energy phonons leads to mode conversion and energy communication between them through phonon scatterings. Thus, the high-energy phonons with low transmission probability will convert to high transmissivity low-energy phonons before they undergo the transport process across the interface, leading to inelastic phonon transport.

For diffuse interfaces, the difference of transmissivity between the low- and high-energy phonons becomes smaller compared with that of sharp interfaces, as the transmissivity for all phonons reduces. As a result, the temperature difference of different phonons is expected to be smaller, thus the phonon non-equilibrium is smaller for diffuse interfaces. The reduced phonon non-equilibrium leads to less energy communication between high and low-energy phonons, and the inelastic phonon transport will diminish.

Our results point out that a large Debye temperature difference is not required for the inelastic phonon transport process to occur, as the Debye temperature ratio of both Al/Si and Al/GaN is <1.5. This finding is in stark contrast to what previous experiments suggested, where inelastic transport can be only observed across interfaces formed with a large Debye temperature difference^12,13,21^.

A recent work by Cheng et al. claimed that no inelastic phonon transport is observed in atomically sharp Al/Al_2_O_3_ interface grown by MBE^6^. We would like to point out that at least five monolayers of Al_2_O_3_ near the interface are distorted, as shown in their TEM image, which is similar to our Al/Si Sample 2 and not as sharp as our Al/Si Sample 1. Their work actually echoes with our finding that significant inelastic thermal transport can only be observed at atomically sharp interfaces.

In applications with high power density, high frequency, and small sizes, such as power electronics and RF devices, heat is mostly localized, leading to hotspots. In such cases, interface heat transport becomes more important^33^. Our work is particularly useful to improve heat dissipation in semiconductor interfaces and metal/semiconductor interfaces at elevated temperatures, especially when the interfaces are formed with low Debye temperature materials such as Au, Ti, and GaAs, as an additional channel across interfaces is open for heat conduction when they are atomically sharp, which is still a technological challenge to date.

In summary, we report the observation of inelastic phonon transport across high-quality Al/Si and Al/GaN interfaces grown by MBE. We observed a continuously increasing thermal conductance at high temperatures, which is attributed to the inelastic phonon transport process across the interface. The inelastic phonon transport is expected to occur at atomically sharp interfaces where the strong phonon non-equilibrium exists, in contrast to diffuse interfaces. This work sheds light on increasing thermal conductance across the interface at high temperatures and improving heat dissipation of electronic devices.

## Methods

### Samples

The epitaxial growth of Al was carried out in a molecular beam epitaxy (MBE) system with a base pressure of 2.7 × 10^−7^ Torr. A Si wafer was cleaned by hydrofluoric acid to remove the native oxide layer before being loaded into the MBE chamber. The Si wafer was degassed at 900 °C for 90 min, and then cooled down for aluminum growth. To form an Al/Si interface with controlled quality, Al growth was proceeded at different temperatures: 100 °C for Sample 1 and 300 °C for Sample 2. The deposition rate of Al was 15 nm/min. On GaN substrate, Al was grown at 150 °C.

### TDTR measurement

We measured interface thermal conductance of Al/Si and Al/GaN at 80–700 K by time-domain thermoreflectance (TDTR) in a Janis VPF-800 cryostat^25^. From 80 to 350 K, the measurement was performed with a 5x objective lens with 1/e^2^ radius of 10.6 µm and a modulation frequency of 10.1 MHz for the pump beam. When the temperature was above 350 K, a 10x objective lens with 1/e^2^ radius of 5.3 µm was used in order to obtain a higher signal-to-noise ratio. We have determined that there is no spot-size-dependent thermal conductance at room temperature in Supplementary Information Note VII. The total laser power is listed in Supplementary Information Note VI, with a steady-state temperature rise within 10 K.

### TEM

HAADF-STEM images were acquired using an aberration-corrected FEI Titan Themis G2 with an acceleration voltage of 300 kV. The convergence semi-angle is 30 mrad, and the collection semi-angle is 39–200 mrad. TEM samples were first thinned by mechanical polishing, and then milled by using a precision ion polishing system with an argon ion source. The acceleration voltage of 4 kV was used until a hole appears. Then accelerating voltage of 0.2 kV was used to remove the amorphous layer.

### DMM calculation

We calculated the interfacial thermal conductance with DMM framework and considered the full phonon dispersion of the materials. Firstly, we obtained the phonon dispersion using density functional theory by Quantum-Espresso package^34^. Generalized gradient approximations (GGA) of Perdew-Burke-Ernzerhof (PBE)^35^ were adopted for exchange-correlation potential. The kinetic energy cutoff for the plane-wave basis was 40 Ry, and a $20\times 20\times 20$ k-point mesh was used to sample the Brillouin zone. The key parameter to calculate the interface thermal conductance is the phonon transmit probability. Consider a phonon mode of materials A with wave vector *k* and polarization *i* that is incident on the interface, the probability that the phonon can transmit from A to B according to DMM is expressed as:

1 $${\alpha }_{A\to B}\left({\omega }^{{\prime} }\right)=\frac{\triangle {K}_{B}\left[\sum \left|V(k,j)\cdot \hat{n}\right|\right]{\delta }_{\omega \left(k,j\right),\omega {\prime} }}{\triangle {K}_{A}\left[{\sum }_{i,k}\left|V(k,i)\cdot \hat{n}\right|\right]{\delta }_{\omega \left(k,i\right),\omega {\prime} }+\triangle {K}_{B}\left[\sum \left|V(k,j)\cdot \hat{n}\right|\right]{\delta }_{\omega \left(k,j\right),\omega {\prime} }}$$

where $\triangle {K}_{A}$ and $\triangle {K}_{B}$ are the volume of the discretized cells pertaining to the high-resolution Brillouin zones of material A and B. The $V$ and $\omega$ are the phonon group velocity and frequency. $\hat{n}$ is the unit vector normal to the interface and *j* is the polarization of phonon in material B. It should be noted that the Kornecher delta function ${\delta }_{\omega \left(k,j\right),\omega {\prime} }$ is unity when the phonon frequencies from the two Brillouin zones are equal and are zeros otherwise. Then the interface thermal conductance can be obtained with the transmission probability according to the Landauer formula, which is given by:

2 $$G=\frac{1}{2{(2\pi )}^{3}}\mathop{\sum}\limits_{i}{\int }_{k}\frac{1}{{k}_{B}{T}^{2}}{\alpha }_{A\to B}(k,i)\times {(\hslash \omega (k,i))}^{2}|V(k,i)\cdot \hat{n}|\times \frac{\exp \left(\frac{h\omega (k,i)}{{k}_{k}T}\right)}{{\left[\exp \left(\frac{\hslash \omega (k,i)}{{k}_{B}T}\right)-1\right]}^{2}}dk$$

### MD simulation

The cross-section area of the simulation system is 19.89 × 22.97 × 10^−20^ m^2^. The simulation was based on non-equilibrium molecular dynamics (NEMD) method, which applied heat bath at both ends of the structure and calculated the interface thermal conductance through:

3 $$G=\frac{J}{A\varDelta T}$$

where *J* is heat flux across the interface at unit time (W), *A* is cross-section area (m^−2^), and $\Delta T$ is temperature drop across the interface (K). The simulation was carried out using LAMMPS^36^ with a time step of 1 fs. The modified embedded atomic method (MEAM) potential of Jelinek et al.^37^ was used to describe Si–Al interaction. This potential was satisfied the Si, Al, and their compounds. The detailed parameters can be obtained from the original reference. The system was first relaxed under canonical ensemble (NVT) for 500 ps. Then the simulation was switched to micro-canonical ensemble (NVE), and the temperatures of two ends of the system were controlled through the Langevin thermostat. This step was carried out for 6 ns to make the system reach a steady state. Additional 6 ns was used to collect the data of the temperature and heat flux to evaluate the interfacial thermal conductance. The structure length L was set as 33 nm, and the structure along the other two dimensions were periodic.

To form a diffuse interface in the simulation, we first defined a region with a certain thickness (1.3 nm), then this region was melted locally at 3000 K, followed by a quick quench to 300 K. And for the interface mismatch, based on our STEM images, we found that the number densities of Si and Al at the interface are about 2.8 and 3.5 1/nm. Our NEMD simulations well reproduced the densities observed in measurements.

### AGF calculation

We used AGF to compute the spectral phonon transmission coefficient from Si side to Al side. Under harmonic approximation, the Green’s function ${G}_{d,d}$ for the interfacial region can be calculated as^38^

4 $${G}_{d,d}={\left[{\omega }^{2}I-{H}_{d,d}-{\it{\Sigma}}_{1}-{\it{\Sigma}}_{2}\right]}^{-1}$$

where $\omega$ is the phonon frequency, ${H}_{d,d}$ is the dynamical matrix of the whole interfacial region, and ${\it{\Sigma}}_{1}$ and ${\it{\Sigma}}_{2}$ are the self-energy matrices of the left (Si) and right (Al) reservoirs. The elements in the dynamical matrices are express as

5 $${H}_{i,j}=\frac{1}{\sqrt{{m}_{R}{m}_{{R}^{{\prime} }}}}\frac{{\partial }^{2}E}{\partial {u}_{R}^{\alpha }\partial {u}_{{R}^{{\prime} }}^{\beta }}$$

where *i (j)* stands for the *i*-th (*j*-th) degree of freedom in the system or the *α (β)* direction of the atom at $R$ (${R}^{{\prime} }$); $m$ is the atomic mass, and *E* is the potential energy of the system. The interatomic potential was also described by the MEAM potential. We evaluated the energy derivatives through the finite difference method by displacing the atoms away from their equilibrium position by 0.01 $\mathring{\rm A}$. The total phonon transmission across the interfacial region is calculated as

6 $$\varXi (\omega )={Tr}[{\varGamma }_{1}{G}_{d,d}{\varGamma }_{2}{G}_{d,d}^{+}]$$

where ${\varGamma }_{1(2)}=i\left({\it{\Sigma}}_{1(2)}-{\it{\Sigma}}_{1(2)}^{+}\right)$, and + means the conjugate transpose of the matrix. The phonon transmission coefficient is defined as the ratio $\varXi (\omega )/M(\omega )$, where $M(\omega )$ is the total number of phonon modes at frequency $\omega$ from Si.

### Reporting summary

Further information on research design is available in the Nature Research Reporting Summary linked to this article.

## Supplementary information

Supplementary Information

Lasing Reporting Summary

Reporting Summary

## Data Availability

Data underpinning the figures that support this work are available within the paper and its Supplementary Information files.

## Code availability

The analysis codes that support the findings of the study are available from the corresponding authors upon reasonable request.
