# Supplementary material for: Inelastic phonon transport across atomically sharp metal/semiconductor interfaces
Source: Nat Commun. 2022 Aug 20;13:4901. doi: 10.1038/s41467-022-32600-w (PMC9392776; doi:10.1038/s41467-022-32600-w)
Supplement: Supplementary file 1 — Supplementary Information [file 41467_2022_32600_MOESM1_ESM.pdf]

Supplementary Information for

Inelastic phonon transport across atomically sharp metal/semiconductor interfaces

Qinshu Li[1,*], Fang Liu[2,3,*], Song Hu[4,*], Houfu Song[1,*], Susu Yang[2], Hailing Jiang[2], Tao Wang[5], Yee Kan Koh[6], Changying Zhao[4], Feiyu Kang[1,7], Junqiao Wu[8,9], Xiaokun Gu[4,†], Bo Sun[1,7,†] and Xinqiang Wang[2,3,†]

[1]Tsinghua-Berkeley Shenzhen Institute, Tsinghua University, Shenzhen 518055, China.

[2]State Key Laboratory for Mesoscopic Physics and Frontiers Science Center for Nano-optoelectronics, School of Physics, Peking University, Beijing 100871, China.

[3]Collaborative Innovation Center of Quantum Matter, Beijing 100871, China.

[4]Institute of Engineering Thermophysics, School of Mechanical Engineering, Shanghai Jiao Tong University, Shanghai 200240, China.

[5]Electron Microscopy Laboratory, School of Physics, Peking University, Beijing 100871, China.

[6]Department of Mechanical Engineering and Center of Advanced 2D Materials, National University of Singapore, 117576 Singapore.

[7]Tsinghua Shenzhen International Graduate School and Guangdong Provincial Key Laboratory of Thermal Management Engineering & Materials, Shenzhen 518055, China.

[8]Department of Materials Science and Engineering, University of California, Berkeley, CA 94720, USA.

[9]Materials Sciences Division, Lawrence Berkeley National Laboratory, Berkeley, CA 94720, USA.

*These authors contributed equally.

[†]To whom correspondence should be addressed: sun.bo@sz.tsinghua.edu.cn, wangshi@pku.edu.cn, xiaokun.gu@sjtu.edu.cn

I. Al THIN FILM GROWTH AND CHARACTERIZATION

A. MBE growth of Al (111) film on Si (111) substrate

The high-energy electron diffraction (RHEED) is used to monitor the growth process of an 80-nm-thick Al film on Si substrate. As shown in Supplementary Fig. 1a, the Si surface has a typical (7×7) reconstructed diffraction patterns after thermal treatment at 900 °C for 30 minutes, indicating that there is no evidence of oxide residue on Si surface[1]. As the epitaxy starts at 100 °C, the stripe patterns of the Al film immediately appeared and remained unchanged. This phenomenon is mainly due to the formation of a sharp epitaxial interface between Al and Si. The X-ray diffraction (XRD) spectrum in Supplementary Fig. 1b shows a sharp peak at 38.4° from Al(111) plane and another sharp peak from Si(111) plane at 28.4°, and the full width at half maximum (FWHM) value of the ω-rocking curves for the Al(111) plane is 0.13°, which further confirms the high crystallinity of the epitaxial Al film[2,3]. Then, the surface morphology of the Al film is characterized by atomic force microscopy (AFM). It is found that the Al film grown at 100 °C (Al/Si Sample 1) has a continuous flat surface with a small root mean square (RMS) roughness of 0.26 nm, as shown in Supplementary Fig. 1c. When the epitaxial temperature rises to 300 °C, the surface morphology of the Al film was deteriorated significantly (Supplementary Fig. 1d), and the RMS roughness of the Al film (Al/Si Sample 2) increases to 17.1 nm. Such large surface roughness in Sample 2 is mainly due to the surface voids, as shown in Supplementary Fig. 1d. If we exclude the contribution from voids, the surface roughness is ~0.7 nm, which is twice as large as that of Sample 1, see subsection D for more details.

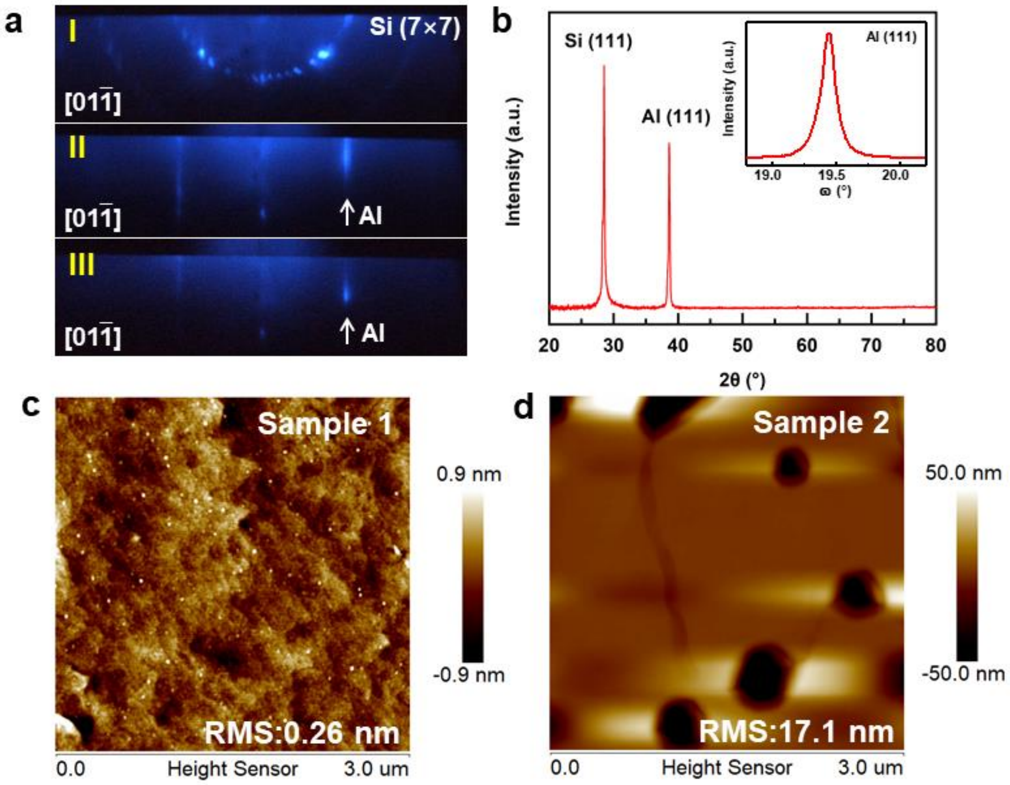

Supplementary Fig. 1 Characterization of Al/Si Sample 1 and Sample 2. (a) In-situ RHEED patterns of the Si(111) substrate just before Al(111) growth (I), after 1-minute growth (II) and after 20-minute growth (III) at 100 °C with the incident electron beam parallel to the [01$\bar{1}$] direction of the Si substrate. (b) XRD 2θ-ω scan and ω-scan spectrum of the epitaxial Al film on Si. (c) AFM image with a 3×3 μm^2 scanned area of Al/Si Sample 1. (d) AFM image with a 3×3 μm^2 scanned area of Al/Si Sample 2.

B. Film thickness

The Al film thickness can be determined from the acoustic echoes in the TDTR signal, with an uncertainty of ~3 nm. Supplementary Fig. 2 shows the acoustic echoes of Al/Si and Al/GaN samples.

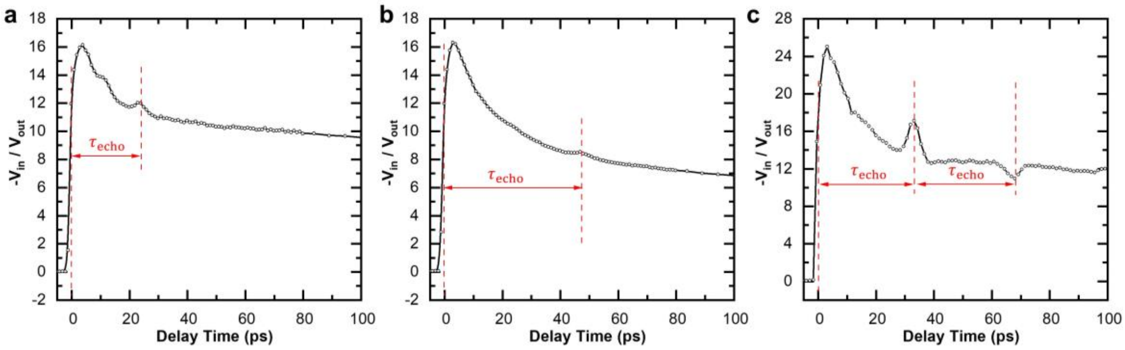

Supplementary Fig. 2 Picosecond acoustic echoes in the TDTR signal. (a) Al/Si Sample 1. (b) Al/Si Sample 2. (c) Al/GaN.

Each echo results from one round trip of thermal stress induced acoustic waves from the surface to the Al/substrate interface. The film thickness can be determined as:

$$t = \frac{1}{2}\tau_{echo}v + 3 \quad (1)$$

where t is the thickness of the Al film, τ_{echo} is the time interval between the echoes. Here for Al/Si samples, τ_{echo} is the time from 0 ps to the first acoustic peak. The v is the longitudinal speed of sound in the Al. We add 3 nm in film thickness in order to account for the additional heat capacity from the native oxide layer.

As shown in Supplementary Fig. 2, τ_{echo} for Al/Si sample 1 is 24 ps, for Al/Si sample 2 is 47 ps and for Al/GaN is 34 ps. The longitudinal sound speed of Al is 6420 m/s. Thus, the thickness of Al film can be calculated and is 80 nm, 153 nm and 112 nm for Al/Si sample 1, sample 2 and Al/GaN, respectively.

C. Electrical resistivity

Four-point probe were used to determine the electrical resistivity of the Al film, which can be calculated as:

$$\rho = 4.532\frac{V}{I}\, t\, k \quad (2)$$

Where ρ is the electrical resistivity, V is the voltage measured between probes 2 and 3, I is the source current, t is the thickness of the Al thin film and k is the correction coefficient.

The measured electrical resistivities are listed in Supplementary Table 1, showing that the two Al/Si samples have similar electrical properties, despite large holes are observed in Al/Si sample 2.

Supplementary Table 1 Measured electrical resistivities of Al/Si samples.

Sample	V/I (Ω)	$(V/I)_{ave}$ (Ω)	t (nm)	k	ρ (Ω m)
Sample 1	0.1111<br>0.1045<br>0.1093	0.1083	80	0.9343	3.669e-8
Sample 2	0.0633<br>0.0560<br>0.0516	0.0570	153	0.9343	3.693e-8

D. Imperfections of the Al layer

Though the Al films are in good quality, we emphasize that there are imperfections that should be taken into consideration.

(1) Domains

High-angle annular dark-field scanning transmission electron microscopy (HAADF-STEM) was used to help identify these imperfections of film. As shown in Supplementary Fig. 3, there are domains

with different orientations in the Al film. The domain size can be identified through HAADF-STEM images, which is at least hundreds of nanometers and much larger than the mean free paths of the electrons and phonons of Al[4]. Besides, according to the electrical resistivity of the Al film in the former section, the suppression of the thermal transport in the Al layer by domains could be negligible.

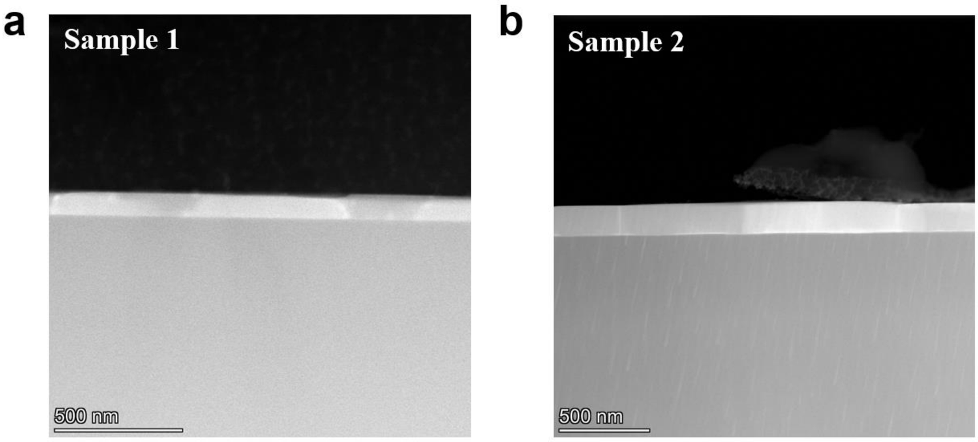

Supplementary Fig. 3 A HAADF-STEM image and a schematic diagram of the Al layer above the Si substrate. (a) Al/Si Sample 1. (b) Al/Si Sample 2.

(2) Voids

Shown in the Supplementary Fig. 1d, at the 3×3 μm² scanned area of Sample 2, the surface roughness is one order larger than that of Sample 1, and there are some voids with around 50 nm depth in the Al film resulting from the high temperature of the Al growth. To further determine its general surface morphology, AFM was performed at some other spots with 5×5 μm² scanned area. As shown in Supplementary Fig. 4a, the RMS roughness is 0.7 nm. This suggests that the large surface roughness in Supplementary Fig. 1d is mainly due to the surface voids, and the surface is rather flat where there are no voids. In order to avoid these voids diffusively scatter probe and pump laser beams in TDTR measurement, we located the laser on a relatively smooth area (Supplementary Fig. 4b). We used a CCD camera to determine the intensity profile of the laser beam, in order to check if the laser beam distribution was distorted by the surface roughness or not. The result is shown in Supplementary Fig. 4c-d, along the vertical line profile shown in Supplementary Fig. 4b, the intensity profile of the laser beam shows Gaussian distribution, which indicates the laser beam is not distorted by the rough surface of Sample 2.

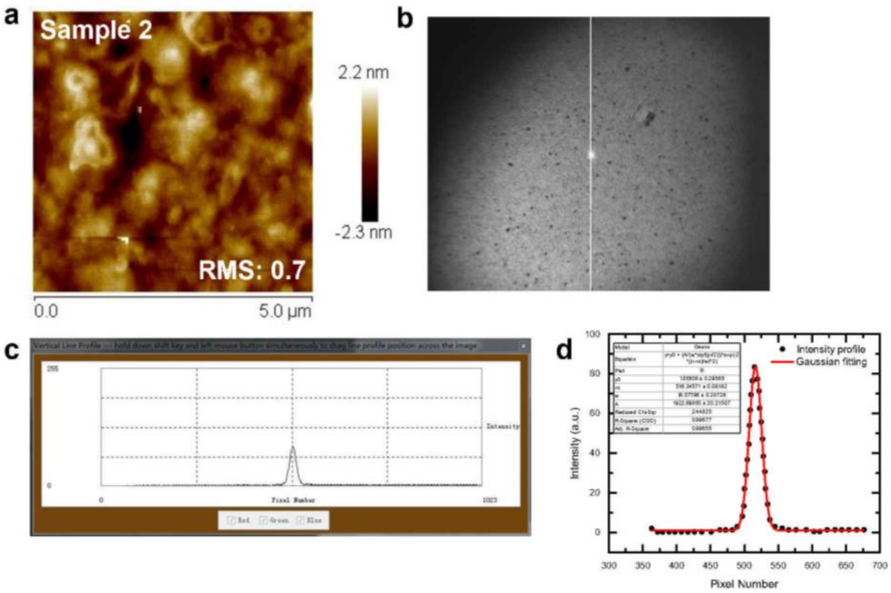

Supplementary Fig. 4 Characterization of surface roughness for Al/Si Sample 2 and the intensity profile of laser located on Sample 2 surface. (a) AFM image with a 5×5 μm^2 scanned area of Al/Si Sample 2. (b) The bright-field microscope image of Sample 2 when TDTR measurements were performed at relatively smooth spots, and (c) the vertical intensity distribution of the laser beam shown in (b). (d) Gaussian function fitting of the vertical intensity profile.

E. Strain at the interface

The geometrical phase analysis (GPA) is an efficient and simple method to measure the local strain in nearly periodic images, which is sensitive to the small displacement of lattice fringes relative to reference lattice. The strain field can be obtained from the displacement filed, thus we can get the strain information[5]. GPA of high-resolution images, such as HAADF-STEM, can map strains at levels of accuracy[6].

Here, the GPA was performed at the Sample 1 interface along the out-of-plane direction in HAADF-STEM (Supplementary Fig. 5a) to characterize the strain. As shown in Supplementary Fig. 5b, the strain map clearly shows abrupt change of the out-of-plane strain component (ε_{yy}) at the exact Al/Si interface, while for both Al film and Si substrate, the ε_{yy} is consistent. And the ε_{yy} strain profile extracted from the strain map is shown in Supplementary Fig. 5c, which further demonstrates that along the white rectangular region in Supplementary Fig. 5b, the ε_{yy} jumps from -0.2% to 0.22% at the interface. And this abrupt variation of the ε_{yy} at the interface may result in the bright contrast area in the STEM image. The GPA map also indicates that the interface strain is localized to the atom layers adjacent to the interface only.

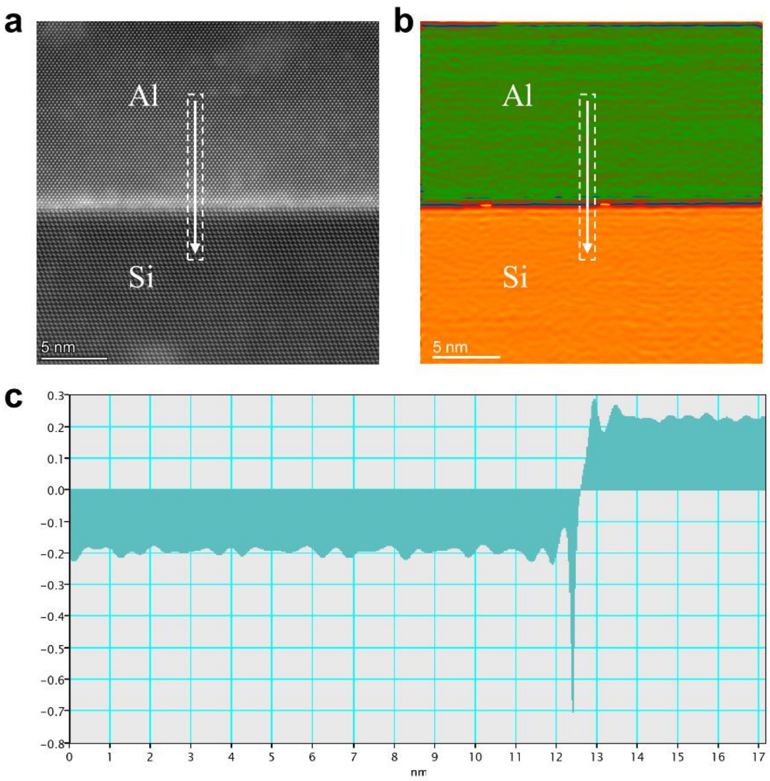

Supplementary Fig. 5 HAADF-STEM and GPA images of interface for strain analysis. (a) HAADF-STEM image of Al/Si Sample 1 interface. (b) Map of out-of-plane strain for the Al/Si interface determined from GPA. (c) ε_{yy} strain profile is extracted from the strain map in the white rectangular region of (b). The white arrow indicates the extraction direction.

II. CHARACTERIZATION OF INTERFACE STRUCTURE OF AL/SI SAMPLES

A. Estimation of interdiffusion region

Low magnitude TEM images (Supplementary Fig. 3) shows the interface is homogeneous, and for a detailed characterization of the interface quality, we introduced interdiffusion depth *d*, defined as the length of interdiffusion region[7], as the quantified parameter for the diffusive interface such as Al/Si Sample 2. For Al/Si Sample 1, the interface is almost coherent and abrupt, we thus used *d* to describe the length of region where atoms are distorted. Firstly, the HAADF-STEM was performed on Al/Si samples to determine the interface atomic structures, shown in the Supplementary Fig. 6. For each sample, we randomly chose 2 spots which were cut off from different parts of the Al/Si wafer. Then we digitized the HAADF-STEM pictures using Cartesian coordinates and determined both the upper and lower bound of the distorted layers. The interdiffusion depth of interface was characterized by the distance between the upper and lower bound.

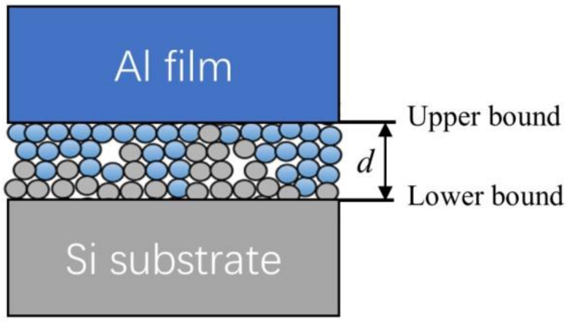

Supplementary Fig. 6 Schematic representation of Al/Si interdiffusion depth *d*, which is the distance between the upper and lower bound of the interdiffusion region intermixed with Al atoms (blue spheres) and Si atoms (grey spheres).

The digitization results are shown in Supplementary Table 2, where the σ_{upper} and σ_{lower} represent the roughness of the upper and lower bound of interdiffusion region respectively. For two spots on the Al/Si Sample 1 interface, *d* are about 0.31 nm and 0.28 nm, which are more or less the same with the interatomic distance between Al and Si atoms across interface. This is why we call Al/Si Sample 1 as "sharp" interface. In the worst-case scenario, both layers adjacent to the interface is distorted. So, for Al/Si Sample 1, less than 2 layers of atoms is distorted. For Al/Si Sample 2, as shown in Supplementary Fig. 7c-f, the average *d* are 1.38 nm and 1.20 nm for two spots respectively, which roughly equals the thickness of 3-5 atomic layers of Si. Here, the interdiffusion depth is up to the wavelength of phonons[4], which is a crucial parameter to affect the phonon transport in this work.

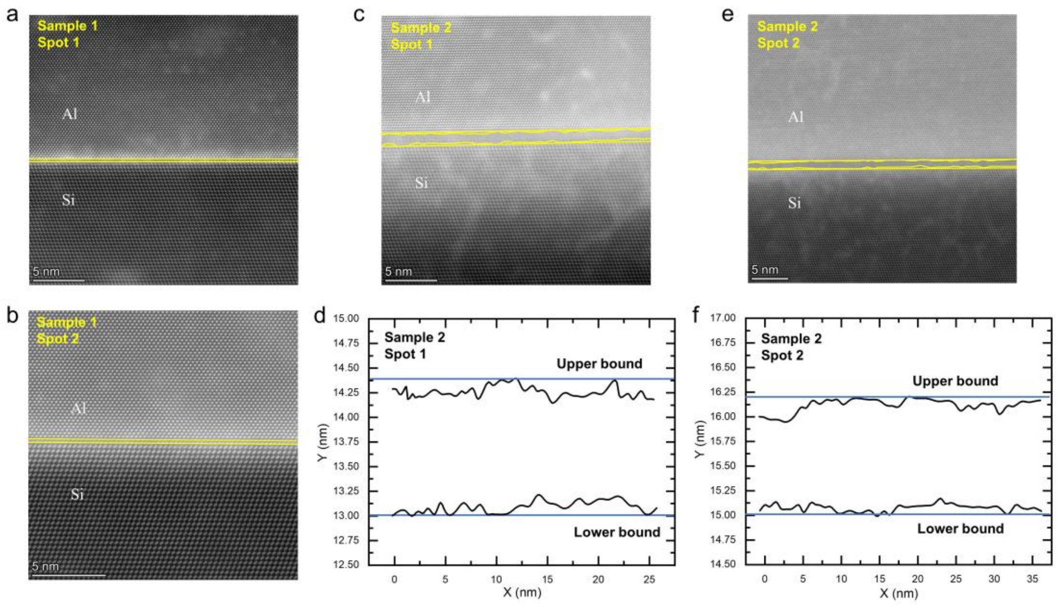

Supplementary Fig. 7 Estimation of the interdiffusion region for Al/Si interfaces. (a) (b) HAADF-STEM image of Al/Si Sample 1 interface. (c) (e) HAADF-STEM image of Al/Si Sample 2 interface. (d) (f) Digitization of the Sample 2 interface. The bound of the distorted layers are denoted in black lines and the upper and lower bound of the interdiffusion region determined are denoted in blue lines.

Supplementary Table 2 Digitization results of Al/Si Sample interfaces.

Spot	σ_{upper} (nm)	σ_{lower} (nm)	d (nm)
Sample 1 spot 1	\	\	0.31
Sample 1 spot 2	\	\	0.28
Sample 2 spot 1	0.14	0.07	1.38
Sample 2 spot 2	0.13	0.11	1.20

In STEM measurements, we can only measure several random spots to determine interface quality. Although these measurements are consistent, questions remain if the interface quality are the same across the whole film. To address this concern, we measured the average interface roughness using the X-Ray reflection spectra (XRR), a reflectometry that can determine interface roughness with mm-sized spot. The reflectivity of a N-layer multilayer system can be calculated based on the Parratt formalism and the roughness put into calculation is used the theory of Nevot and Croce[8], thus the interface roughness here can be defined as Nevot Croce roughness. The parameters for theoretical simulations and the results are shown in Supplementary Table 3, where t represents the layer thickness, ρ represents the mass density,

β and δ are the real and imaginary parts of the refractive index of the layer, E represents the electron density.

Supplementary Table 3 Parameters as the results for data fitting by theoretical simulations.

	t (nm)	ρ (g cm^{-3})	β (×1e8)	δ (×1e8)	E (Å^3)
Al film	80 (Sample 1)<br>153 (Sample 2)	2.71	850.14	16.15	0.79866
Si substrate	430000	2.328	756.86	17.3	0.71102

There are two parameters to fit the measured XRR curves, the surface roughness σ_1 and the interface roughness σ_2. In our case, σ_1 have been measured using AFM, so we only use σ_2 to fit the XRR curves. The fitted Al/Si interface roughness σ_2 is shown in Supplementary Fig. 8. The interface roughness for Al/Si Sample 1 is 0.5 nm, which is in good agreement with interdiffusion depth d determined using STEM. For Al/Si Sample 2, we can hardly fit the measured XRR curve as there's no clear oscillations shown as Supplementary Fig. 8b. Such discrepancy between the measured XRR curve and fitting data is not rare and has been studied extensively before[8], which could result from the lack of consideration of diffusive scattering of the refracted x-rays and the effective roughness depending on the incident angle, especially for a surface and interface with a large degree of randomness[9–11]. So, for a more accurate measurement of interface quality, we still use STEM data on randomly chosen spots in the Al/Si wafer.

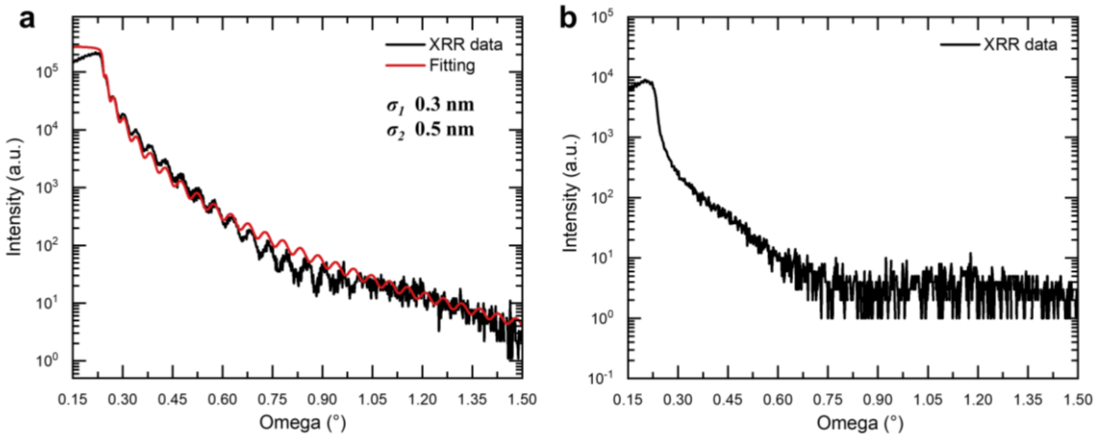

Supplementary Fig. 8 Measured x-ray reflectivity and fitting data for Al/Si samples. (a) Al/Si Sample 1. (b) Al/Si Sample 2.

B. Characterization of interdiffusion region for Al/Si Sample 2

For Al/Si Sample 2, the interdiffusion region with nearly disordered lattice arrangement between the epilayer and the substrate is obviously thicker than Sample 1, then energy-dispersive x-ray spectroscopy (EDX) was performed and further reveals this interdiffusion region is composed of Si and Al atoms (in Supplementary Fig. 9c and Supplementary Fig. 9d), where Si atoms are enriched on the

side close to the substrate and Al atoms are enriched on the side close to the epilayer shown as the result of EDX line scan analysis (in Supplementary Fig. 9b). This indicates that the interdiffusion region is most likely originated from the intermixing of Al and Si atoms at a higher growth temperature, resulting in a diffuse interface[7]. Note that EDX measurements only give a quantitative analysis due to limited spatial resolution.

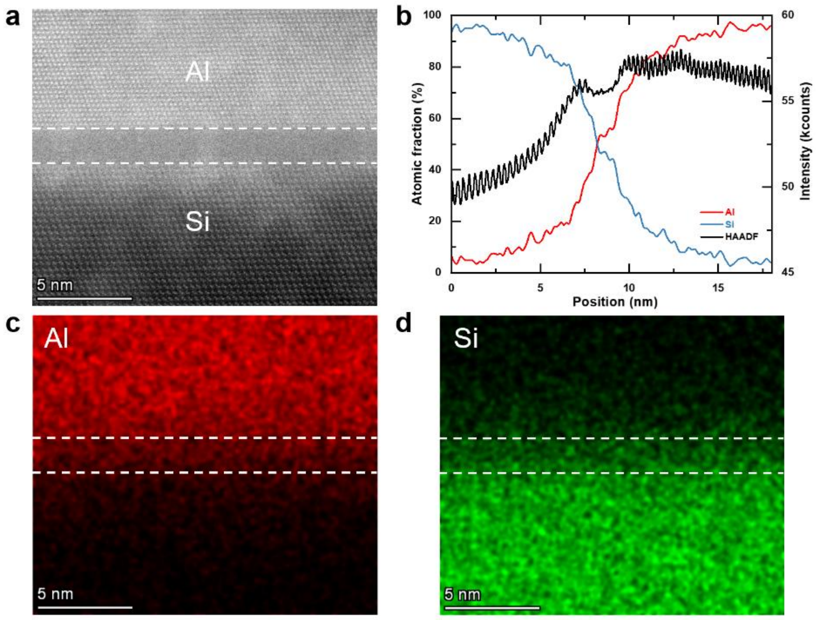

Supplementary Fig. 9 Characterization of interdiffusion region for Al/Si Sample 2. (a) HAADF-STEM image of Al/Si Sample 2 interface. (b) EDX line scan analysis of Al/Si interface, showing the Al and Si atomic fraction at the interfacial region. (c-d) EDX mapping images taken at the Al/Si interface.

To check whether the interfaces would change with temperature, we should better know the interdiffusion rate across Al/Si interface. Interdiffusion across interfaces is always a concern, especially when the interface is heated up. However, previous theoretical and experimental studies showed the dissolution rate is very limited for Al(111) on Si (111). Hellman et al.[12] pointed out that the dissolution rate is zero in the case of (111) epitaxial aluminum on (111) silicon, as there is only a single aluminum site adjacent to silicon surface sites based on a vacancy mediated interdiffusion model. This model is in good agreement with experiments reported by Yamada et al[13]. They found that the surface and interface of Al(111) on Si(111) did not have annealing hillock nor alloy penetration (a sign of dissolution of Si in Al) after 30-min annealing at 450 °C (723.15 K). Their sample was prepared with substrate temperature less than 150 °C.

III. THE CALCULATED G_{PP} FOR AL/SI SAMPLE 1, SAMPLE 2 AND AL/GAN INTERFACES

To calculate the phonon-phonon transport induced interface thermal conductance alone, we follow Majumdar and Reddy's treatment of electron-phonon coupling[14] and use $G = \frac{G_{ep}G_{pp}}{G_{ep}+G_{pp}}$, explained in detail in the main text.

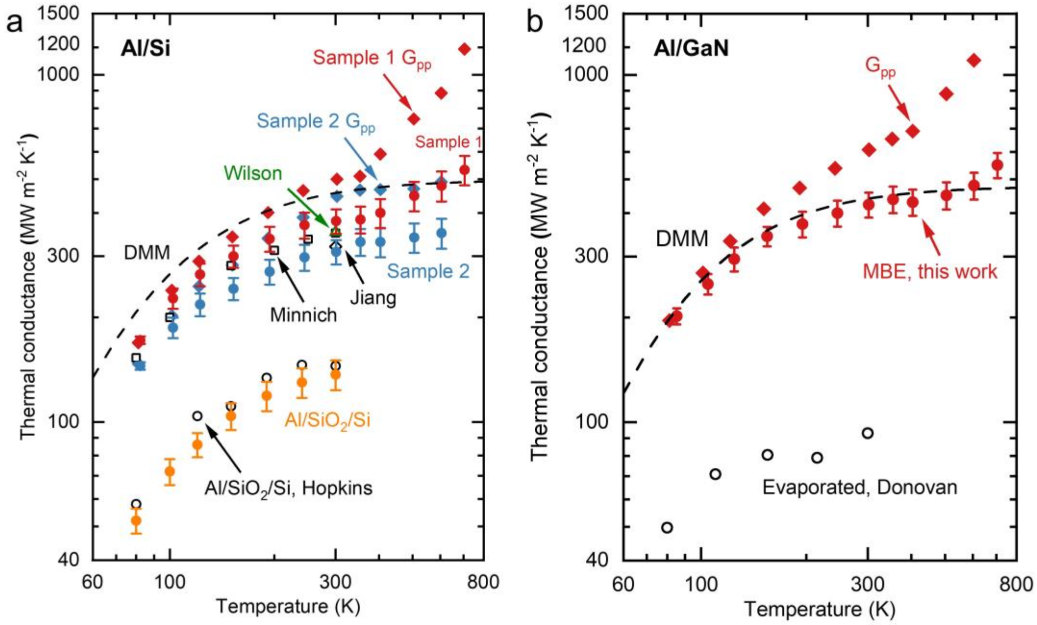

Supplementary Fig. 10 Thermal conductance of Al/Si and Al/GaN interfaces. (a) The calculated G_{pp} of Al/Si Sample 1 (red diamonds) and Sample 2 (blue diamonds). The measured thermal conductance of Al/Si Sample 1 (red spheres) and Al/Si Sample 2 (blue spheres). (b) The calculated G_{pp} of Al/GaN (red diamonds). The measured thermal conductance of Al/GaN (red spheres).

IV. THERMAL CONDUCTANCE OF AL/SI INTERFACES WITH N-TYPE AND INTRINSIC SILICON

We measured the thermal conductance of Al/Si interfaces with both n-type Si and intrinsic Si from 80 K to 700 K under the same experimental conditions. The n-type Si is dopped with As, and the electrical resistivity is ~ 5×10^{-5} ohm m. The electrical resistivity of intrinsic Si is larger than 100 ohm m. The results of measured thermal conductance of the two samples are nearly identical, indicating that the light doping[15] of the substrate could be negligible to the behavior of the phonon transport across interfaces.

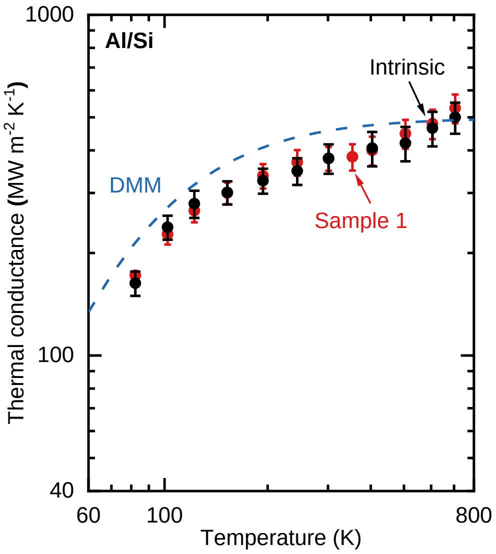

Supplementary Fig. 11 Thermal conductance of Al/Si interfaces from 60 K to 800 K. The measured results of Al/intrinsic-Si are in black spheres, and results of Al/n-type-Si (Sample 1) are in red spheres. For comparison, the calculated thermal conductance of the Al/Si interface by DMM is shown in the blue dash line. The measured results of the two samples are nearly identical.

V. RAW TDTR DATA FOR AL/SI AND AL/GAN SAMPLES

TDTR raw data of Al/Si and Al/GaN samples from 80 K to 700 K are presented in Supplementary Fig. 12 and Supplementary Fig. 13. Here, G_1 and G_2 represent the measured thermal conductance of Sample 1 and Sample 2 respectively.

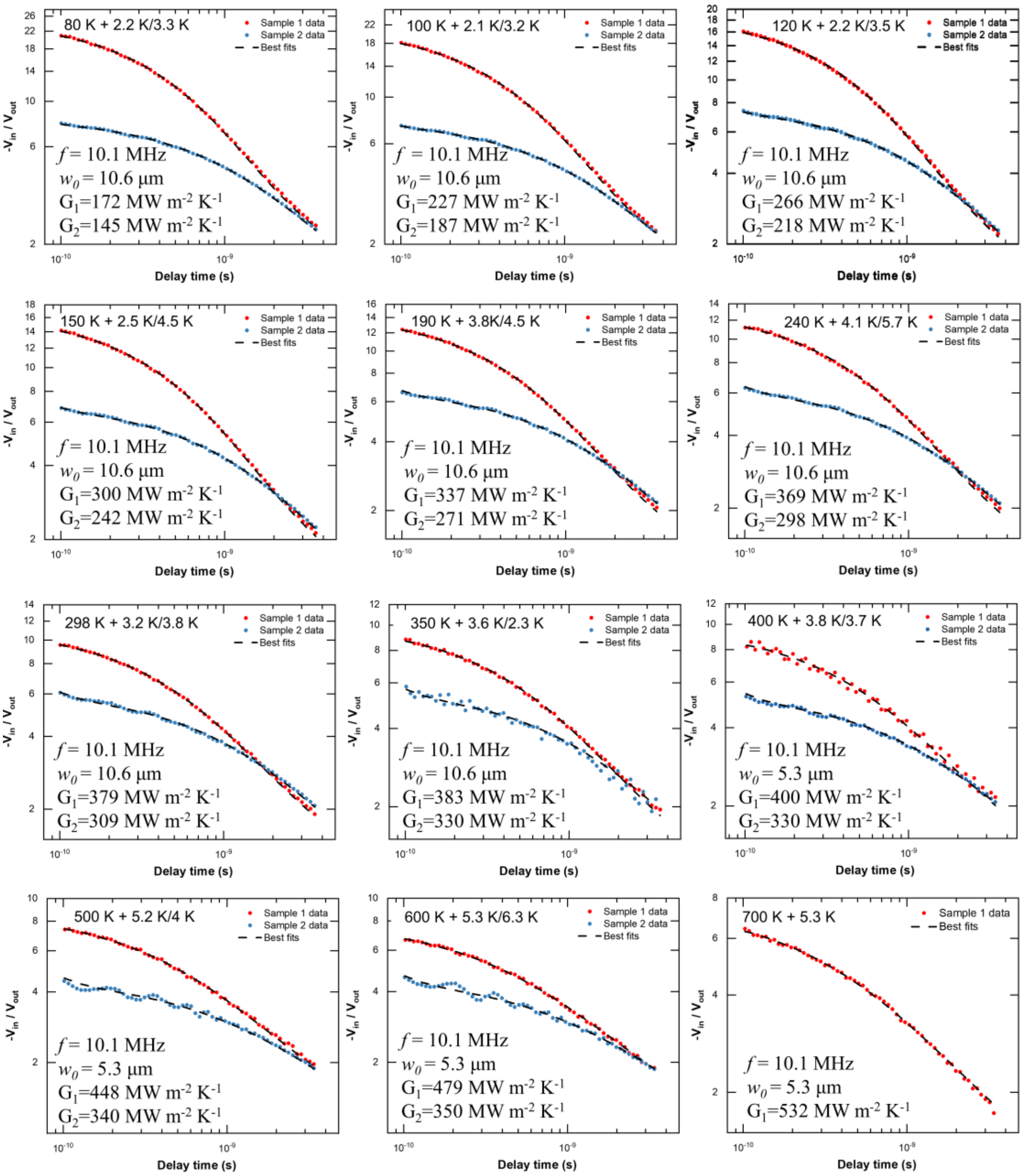

Supplementary Fig. 12 TDTR raw data from 80 K to 700 K for Al/Si Sample1 and Sample 2. For low temperatures, the measurement was performed with 5x objective lens with $1/e^2$ radius of 10.6 μm and a modulation frequency of 10.1 MHz. When the temperature is above 350 K, 10x objective lens with $1/e^2$ radius of 5.3 μm is used in order to obtain better signal to noise ratio.

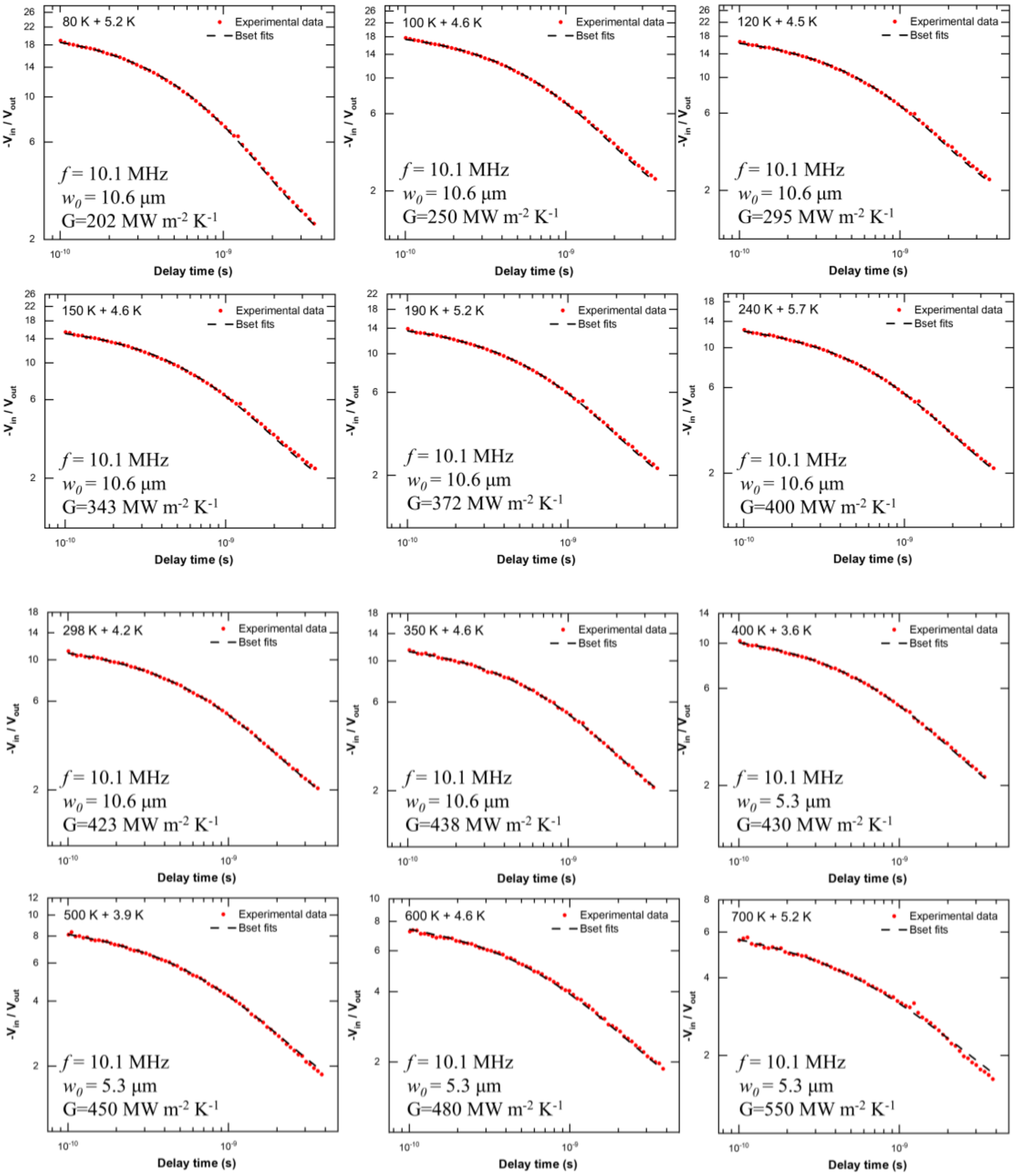

Supplementary Fig. 13 TDTR raw data from 80 K to 700 K for Al/GaN Sample. For low temperatures, the measurement was performed with 5x objective lens with $1/e^2$ radius of 10.6 μm and a modulation frequency of 10.1 MHz. When the temperature is above 350 K, 10x objective lens with $1/e^2$ radius of 5.3 μm in order to obtain better signal to noise ratio.

In Supplementary Fig. 12, we noticed that there were long lived oscillations in the TDTR signal of Al/Si sample 2 and the oscillation period is around 190 ps. These are probably due to the Lamb wave (plate acoustic wave) propagation in Al film, which travels both in-plane and out-of-plane. Wang and Cahill[16] found that in-plane Lamb wave could affect TDTR signal. They found TDTR signal $-V_{in}/V_{out}$ was suppressed until ~200, 500, and 800 ps for beam spot sizes of 1.08, 2.7, and 5.2 μm, respectively,

and hypothesized that the lateral stress in the Al film generated by the pump beam could be relaxed by the propagation of an acoustic wave that was related to the zero-order symmetric Lamb mode (S_0 mode) of the thin Al film. As different spot sizes generate different lateral stress distribution, they observed spot size dependent relaxation. However, the period we observed was spot size independent shown as Supplementary Fig. 14, suggesting the relaxation of thermoelastic stresses were the same with different spot sizes. Besides, the oscillations coming from Lamb wave travelling in-plane and reflected back by boundaries set by deep holes in Al film of Sample 2, which are tens to hundreds of microns apart (see Supplementary Fig. 4b the optical image), correspond to a group velocity on the order of 10^5-10^6 m/s, which was too high for a Lamb wave. Thus, we believe the periodic oscillation in our measurements were from out-of-plane Lamb wave propagation rather than in-plane Lamb wave.

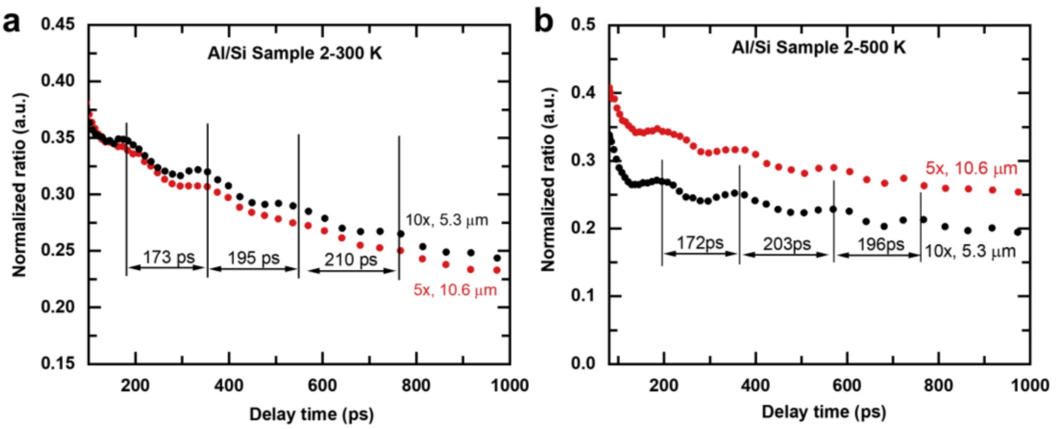

Supplementary Fig. 14 Oscillations of TDTR signal along delay time of Al/Si Sample 2 with different spot sizes at 300 K and 500 K. (a) At 300 K. (b) At 500 K. The measurement was conducted using a 10x objective lens with $1/e^2$ radius of 5.3 μm (black spheres) and a 5x objective lens with $1/e^2$ radius of 10.6 μm (red spheres).

Here, the superposition of reflected out-of-plane Lamb wave by Al/Si interface and the original Lamb wave generated by thermal stress leads to clear Lamb wave echos. Considering that there is a phase shift of π when lamb wave reflected at Al/air interface, every two rounds of traveling in Al films leads to a constructed interference. Thus, the Lamb wave has a frequency of ~10 GHz (2/190 ps) and a group velocity of ~3200 m/s (two rounds travel 4×150 nm/190 ps), which suggests they were the zero-order antisymmetric Lamb mode (A_0 mode).

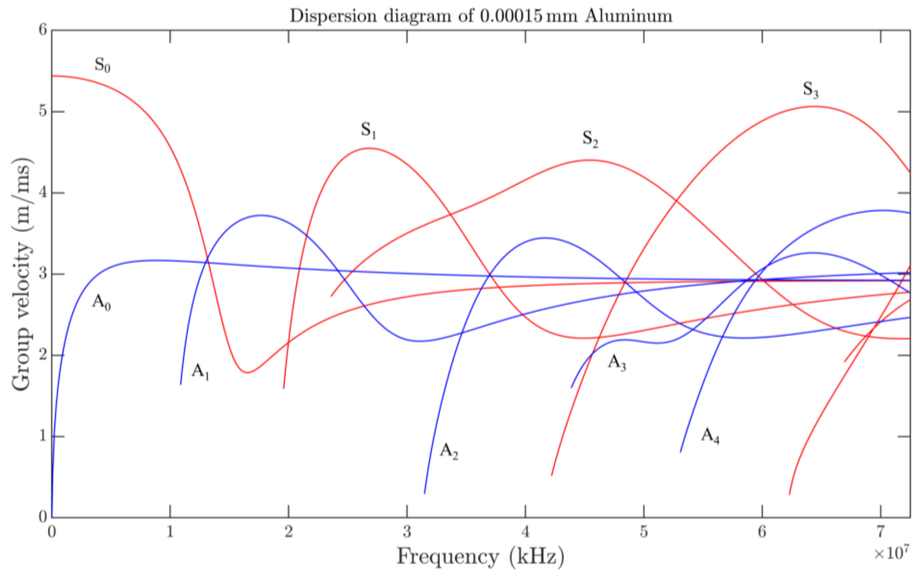

Supplementary Fig. 15 Group velocity dispersion diagram for Lamb waves of a 150 nm-thickness Al layer. The symmetric Lamb modes (S mode) are denoted in red lines, and antisymmetric Lamb modes (A mode) are denoted in blue lines.

VI. PARAMETERS FOR TDTR MEASUREMENTS

The parameters used for TDTR are listed in Supplementary Table 4 to 6, where T is the set temperature of cryostat, *P_pump* is the laser power for pump beam, *P_probe* is the power for probe beam, ΔT is the steady-state temperature rise. C_{Al}, C_{Si}, C_{GaN} is the heat capacity of Al, Si and GaN, h_{Al} is the film thickness of Al, Λ_{Al} is the thermal conductivity of Al thin film, w_0 is the spot size, f is the modulation frequency. Here, the power is measured by a powermeter placed in front of the objective lens with 80% transmittance of the light. For the cryostat window made of quartz, the transmittance of 787 nm laser is 90%. Thus, the total power arrives at the sample surface is the measured power times 72%. At room temperature, with a mm-sized spot, the measured reflectivity for Sample 1 is 81% and is 75% for Sample 2, where the voids in the Al layer would lead to more diffuse reflection.

Supplementary Table 4 Parameters for measurement of Al/Si Sample 1.

T (K)	*P_pump* (mW)	*P_probe* (mW)	ΔT (K)	C_{Al} (J cm^{-3} K^{-1})	C_{Si} (J cm^{-3} K^{-1})	h_{Al} (nm)	Λ_{Al} (W m^{-1} K^{-1})	w_0 (µm)	f (MHz)
700	40	20	5.3	2.89	2.01	80	198	5.3	10.1
600	40	20	5.3	2.78	1.97	80	197	5.3	10.1
500	40	20	5.2	2.69	1.93	80	193	5.3	10.1
400	40	20	3.8	2.58	1.82	80	189	5.3	10.1
350	80	40	3.6	2.52	1.76	80	186	10.6	10.1
298	120	80	3.2	2.43	1.67	80	182	10.6	10.1
240	120	80	4.1	2.29	1.49	80	175	10.6	10.1
190	120	80	3.8	2.11	1.26	80	168	10.6	10.1
150	120	80	2.5	1.86	1.01	80	160	10.6	10.1
120	120	80	2.2	1.58	0.78	80	150	10.6	10.1
100	120	80	2.1	1.33	0.62	80	140	10.6	10.1
80	120	80	2.2	1.02	0.45	80	127	10.6	10.1

Supplementary Table 5 Parameters for measurement of Al/Si Sample 2.

T (K)	*P_pump* (mW)	*P_probe* (mW)	ΔT (K)	C_{Al} (J cm^{-3} K^{-1})	C_{Si} (J cm^{-3} K^{-1})	h_{Al} (nm)	Λ_{Al} (W m^{-1} K^{-1})	w_0 (µm)	f (MHz)
600	118	11	6.3	2.78	1.97	153	197	5.3	10.1
500	70	11	4.0	2.69	1.93	153	193	5.3	10.1
400	58	10	3.7	2.58	1.82	153	189	5.3	10.1
350	80	40	2.3	2.51	1.75	153	186	10.6	10.1
298	120	80	3.8	2.43	1.67	153	182	10.6	10.1
240	200	100	5.7	2.30	1.49	153	175	10.6	10.1
190	200	100	4.5	2.11	1.27	153	168	10.6	10.1
150	200	100	4.5	1.88	1.02	153	160	10.6	10.1
120	200	100	3.5	1.60	0.79	153	150	10.6	10.1
100	200	100	3.2	1.35	0.63	153	140	10.6	10.1
80	200	100	3.3	1.03	0.47	153	127	10.6	10.1

Supplementary Table 6 Parameters for measurement of Al/GaN.

T (K)	*P_pump* (mW)	*P_probe* (mW)	ΔT (K)	C_{Al} (J cm^{-3} K^{-1})	C_{GaN} (J cm^{-3} K^{-1})	h_{Al} (nm)	Λ_{Al} (W m^{-1} K^{-1})	w_0 (µm)	f (MHz)
700	155	22	5.2	2.89	3.4	112	198	5.3	10.1
600	135	20	4.6	2.78	3.30	112	197	5.3	10.1
500	110	20	3.9	2.69	3.18	112	193	5.3	10.1
400	100	20	3.6	2.58	2.95	112	189	5.3	10.1

350	150	70	4.6	2.52	2.83	112	186	10.6	10.1
298	150	70	4.2	2.43	2.63	112	182	10.6	10.1
240	260	120	5.7	2.30	2.30	112	175	10.6	10.1
190	260	120	5.2	2.11	1.95	112	168	10.6	10.1
150	260	120	4.6	1.87	1.58	112	160	10.6	10.1
120	260	120	4.5	1.61	1.27	112	150	10.6	10.1
100	260	120	4.6	1.36	1.10	112	140	10.6	10.1
80	260	120	5.2	1.06	0.75	112	127	10.6	10.1

VII. FREQUENCY AND SPOT SIZE INDEPENDENCE OF G IN TDTR MEASUREMENTS AT HIGH TEMPERATURES

We used both 10x and 5x objective lens to measure the Al/Si samples and Al/GaN at room temperature. Shown in the Supplementary Table 7, the thermal conductance measured using 10x objective lens with $1/e^2$ radius of 5.3 μm is within ~ 4% of values measured by 5x objective lens with $1/e^2$ radius of 10.6 μm.

Supplementary Table 7 G with different laser spot size (G_5x for 10.6 μm and G_10x for 5.3 μm) under the modulation frequency of 10.1 MHz at room temperature.

	G_5x ($MW\ m^{-2}\ K^{-1}$)	G_10x ($MW\ m^{-2}\ K^{-1}$)
Al/Si Sample 1	379	370
Al/Si Sample 2	309	320
Al/GaN	423	415

For high temperatures such as 500 K and 600 K, measurement was also performed using low modulation frequency, in order to increase the amplitude of the signal and the signal to noise ratio. As shown in Supplementary Table 8, the results of thermal conductance are in a good agreement when using 10.1 MHz and 1.01 MHz modulation frequencies. (10.1 MHz with spot size of 5.3 um and 1.01 MHz with spot size of 10.6 um.)

Supplementary Table 8 G with different modulation frequencies at high temperature.

	Al/Si Sample 2		Al/GaN	
	G_10.1 MHz ($MW\ m^{-2}\ K^{-1}$)	G_1.01 MHz ($MW\ m^{-2}\ K^{-1}$)	G_10.1 MHz ($MW\ m^{-2}\ K^{-1}$)	G_1.01 MHz ($MW\ m^{-2}\ K^{-1}$)
500 K	340	360	450	445
600 K	350	370	480	500

VIII. UNCERTAINTY ANALYSIS

Before TDTR measurement, we did the sensitivity analysis to determine the optimized parameters. The sensitivity is defined as,

$$S_\alpha = \frac{\partial(-\frac{V_{in}}{V_{out}})}{\partial \ln\alpha} \quad (3)$$

where $-V_{in}/V_{out}$ is TDTR signal, and α is the parameter that is used in our thermal model. In a typical measurement, there are usually two unknown parameters, interface thermal conductance between transducer and substrate (G) and thermal conductivity of the substrate (Λ). Supplementary Table 9 to S11 shows the calculated sensitivity to all parameters in our thermal model, where h_{Al}, Λ_{Al}, C_{Al}, C_{Si}, w_0 and ϕ are the thickness, thermal conductivity and heat capacity of Al, heat capacity of Si, laser spot radius, and phase of the lock-in amplifier, respectively.

Supplementary Table 9 Sensitivity to the parameters in TDTR measurement of thermal conductance of Al/Si Sample 1 interface.

T	$S_{h_{Al}}$	$S_{\Lambda_{Al}}$	$S_{C_{Al}}$	$S_{C_{Si}}$	$S_{G_{Al/Si}}$	S_{w_0}	S_ϕ
700 K	-0.765	-0.055	-0.858	+0.437	-0.340	-0.061	+7.045
600 K	-0.780	-0.050	-0.870	+0.434	-0.320	-0.070	+7.633
500 K	-0.790	-0.048	-0.880	+0.433	-0.325	-0.075	+8.499
400 K	-0.799	-0.047	-0.882	+0.433	-0.327	-0.080	+9.111
350 K	-0.813	-0.038	-0.893	+0.426	-0.366	-0.085	+10.229
298 K	-0.817	-0.038	-0.894	+0.402	-0.386	-0.112	+10.972
242 K	-0.825	-0.037	-0.894	+0.398	-0.429	-0.114	+12.709
190 K	-0.831	-0.036	-0.897	+0.342	-0.489	-0.174	+14.171
150 K	-0.840	-0.033	-0.905	+0.304	-0.556	-0.214	+17.557
120 K	-0.856	-0.029	-0.911	+0.170	-0.635	-0.354	+22.145
100 K	-0.860	-0.025	-0.915	+0.188	-0.710	-0.339	+26.238
80 K	-0.872	-0.019	-0.923	+0.066	-0.800	-0.467	+33.840

Supplementary Table 10 Sensitivity to the parameters in TDTR measurement of thermal conductance of Al/Si Sample 2 interface.

T	$S_{h_{Al}}$	$S_{\Lambda_{Al}}$	$S_{C_{Al}}$	$S_{C_{Si}}$	$S_{G_{Al/Si}}$	S_{w_0}	S_ϕ
600 K	-0.904	-0.010	-0.990	+0.355	-0.270	-0.086	+4.722
500 K	-0.956	-0.010	-1.040	+0.348	-0.307	-0.089	+4.627
400 K	-0.920	-0.010	-1.010	+0.350	-0.301	-0.090	+5.200
350 K	-0.968	-0.010	-1.000	+0.377	-0.309	-0.032	+5.972
298 K	-1.000	-0.010	-1.035	+0.383	-0.324	-0.032	+6.167
242 K	-0.967	-0.001	-1.000	+0.381	-0.336	-0.038	+6.459
190 K	-0.997	-0.001	-1.030	+0.379	-0.313	-0.053	+6.849
150 K	-0.940	-0.014	-0.977	+0.382	-0.310	-0.065	+7.143
120 K	-0.984	-0.018	-1.022	+0.355	-0.310	-0.113	+7.928
100 K	-0.856	-0.022	-0.902	+0.329	-0.410	-0.149	+8.125
80 K	-0.876	-0.031	-0.923	+0.278	-0.362	-0.210	+8.420

Supplementary Table 11 Sensitivity to parameters in TDTR measurement of thermal conductance of Al/GaN interface.

T	$S_{h_{Al}}$	$S_{\Lambda_{Al}}$	$S_{C_{Al}}$	$S_{C_{GaN}}$	$S_{G_{Al/GaN}}$	S_{w_0}	S_ϕ
700 K	-0.950	-0.009	-1.020	+0.403	-0.300	-0.070	+5.830
600 K	-0.969	-0.015	-1.030	+0.392	-0.298	-0.070	+7.630
500 K	-1.000	-0.040	-1.030	+0.397	-0.310	-0.072	+8.519
400 K	-0.980	-0.020	-1.000	+0.430	-0.310	-0.074	+8.943
350 K	-0.960	-0.030	-0.980	+0.430	-0.300	-0.030	+11.190
298 K	-0.930	-0.020	-0.960	+0.434	-0.312	-0.020	+11.458
242 K	-0.890	-0.020	-0.910	+0.430	-0.300	-0.020	+12.838
190 K	-0.834	-0.030	-0.862	+0.440	-0.310	-0.030	+14.151
150 K	-0.754	-0.014	-0.784	+0.414	-0.380	-0.030	+16.092
120 K	-0.655	-0.015	-0.686	+0.440	-0.427	-0.036	+17.308
100 K	-0.550	-0.016	-0.580	+0.410	-0.470	-0.040	+18.325
80 K	-0.366	-0.020	-0.400	+0.370	-0.550	-0.069	+19.352

After all the sensitivity parameters are calculated, the measurement uncertainty of interface thermal conductance of Al/Si and Al/GaN interfaces can be calculated using the following equation:

$$\left(\frac{\Delta G}{G}\right)^2 = \sum\left(\frac{S_\alpha}{S_G}\frac{\Delta\alpha}{\alpha}\right)^2 + \left(\frac{S_\phi}{S_G}\Delta\phi\right)^2 \quad (4)$$

The uncertainties we used for the parameters are shown in the following Table.

Supplementary Table 12 Uncertainties for the parameters used in our thermal model.

$\frac{\Delta\alpha}{\alpha}$	uncertainty
$\frac{\Delta h_{Al}}{h_{Al}}$	±2%
$\frac{\Delta\Lambda_{Al}}{\Lambda_{Al}}$	±10%
$\frac{\Delta C_{Al}}{C_{Al}}$	±2%
$\frac{\Delta C_{Si}}{C_{Si}}$	±2%
$\frac{\Delta C_{GaN}}{C_{GaN}}$	±2%
$\frac{\Delta w_0}{w_0}$	±10%
$\Delta\phi$	0.001

Put the uncertainties in Supplementary Table 12 into Eq. 4, we then calculated the measurement uncertainty of G, which is shown in our Fig. 1 in the main text.

IX. MD SIMULATION

Supplementary Table 13 Simulated result of thermal conductance as a function of temperature by MD. *dT* is temperature difference of heat bath, and *ΔT* is simulated temperature drop across interface obtained from linear fit.

Interface	Length L (nm)	Heat bath dT (K)	Temperature T (K)	Temperature drop ΔT (K)	Heat flux J (ev ps^{-1})	Thermal conductance G (MW m^{-2} K^{-1})
			300	63.64	0.90	623
sharp	33	60	500	48.00	0.73	738
			700	35.23	0.57	826
			300	57.42	1.02	496
diffuse	33	60	500	39.43	0.83	533
			700	28.78	0.67	567

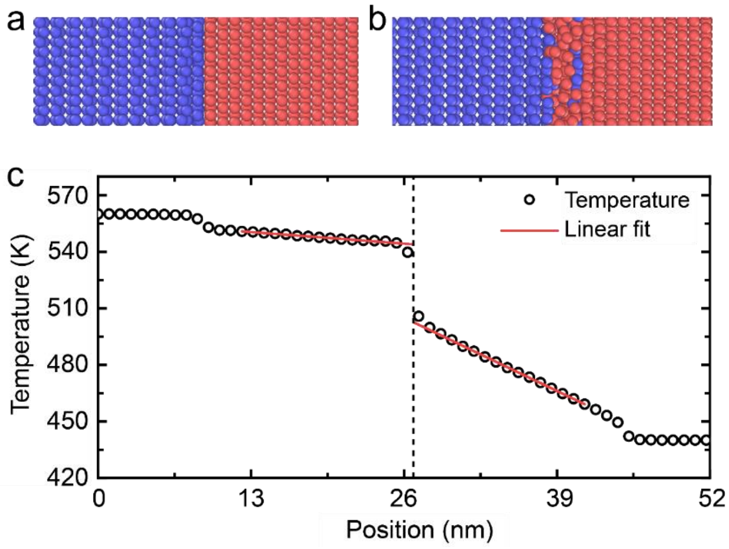

Supplementary Fig. 16 Computational geometry configurations and the temperature profile of rectangle-shaped Al/Si interfaces. (a) A rectangle-shaped with a sharp configuration of Al/Si interface and (b) a diffuse configuration of Al/Si interface. (c) An example of the simulated temperature profile of the sharp interface at 500 K with a length of 33 nm and heat bath temperature difference of 60 K.

X. RELATIONS BETWEEN INTERFACE PROPERTIES AND PHONON TRANSMISSIONS

For the realistic three-dimensional interfaces, the atomic bonding is not necessarily a decisive factor on the phonon transmission compared to a simple one-dimensional model[17]. Especially in our diffuse interface structure (Al/Si Sample 2), the atoms are arranged less regularly. For the sharp interface, the energy transferring across interface must be between Al and Si pairs. However, for the diffuse interface structures, the energy transferring is not necessarily through Al-Si bond only. We evaluate the capability of energy transferring by the participation rate[18]. Supplementary Fig. 17 shows the calculated participation rate of phonons in the interface region for different interface structures. The phonons in the sharp interface are of higher participation ratio than the diffuse one. The low participation rate means the phonon energy is local and have less contribution to heat transfer. Therefore, compared with the sharp one, phonon transmission through the diffuse interface is smaller.

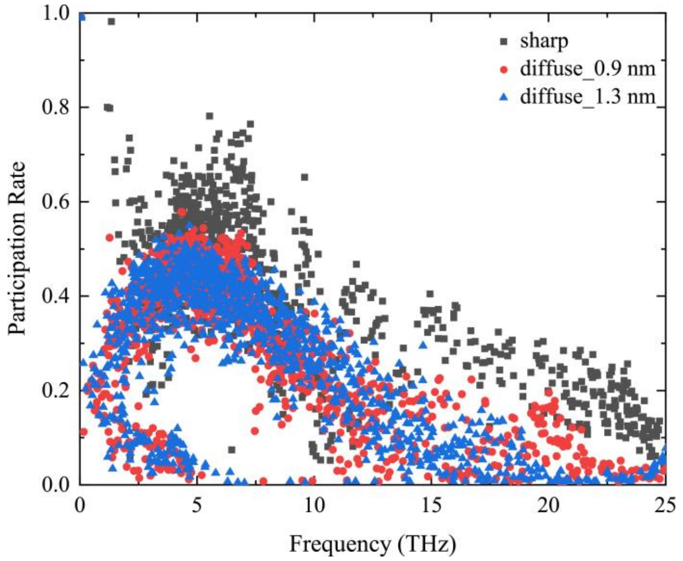

Supplementary Fig. 17 The participation rate of phonons in the interface region for sharp and diffuse interfacial structures. The sharp interface is denoted in black diamonds, the diffuse interface with a 0.9 nm interdiffusion depth is in red spheres, and the diffuse interface with a 1.3 nm diffusion depth is in blue triangles.

The bond stiffness between the atoms in the interfacial region is examined by calculating their spring constants, which are defined as the trace of the harmonic force constant tensor of the neighboring atom pairs. Our calculation shows that the spring constants of the diffuse interface are rather scattered and are larger than those of the sharp interface on average. Apart from the bonding stiffness, the atomic configuration should be a critical factor, as the vibrational properties are highly sensitive to the arrangement of atoms.

Supplementary References

1. Liu, S. *et al.* Molecular beam epitaxy of single-crystalline aluminum film for low threshold ultraviolet plasmonic nanolasers. *Appl. Phys. Lett.* **112**, 231904 (2018).
2. Cheng, F. *et al.* Epitaxial Growth of Atomically Smooth Aluminum on Silicon and Its Intrinsic Optical Properties. *ACS Nano* **10**, 9852–9860 (2016).
3. Chou, Y. H. *et al.* High-Operation-Temperature Plasmonic Nanolasers on Single-Crystalline Aluminum. *Nano Lett.* **16**, 3179–3186 (2016).
4. Jain, A. & McGaughey, A. J. H. Thermal transport by phonons and electrons in aluminum, silver, and gold from first principles. *Phys. Rev. B* **93**, 081206 (2016).
5. Hÿtch, M. J., Putaux, J. L. & Pénisson, J. M. Nanoscale Measurement of Stress and Strain by Quantitative High-Resolution Electron Microscopy. *Mater. Sci. Forum* **482**, 39–44 (2005).
6. Chung, J., Lian, G. & Rabenberg, L. Practical and Reproducible Mapping of Strains in Si Devices Using Geometric Phase Analysis of Annular Dark-Field Images From Scanning Transmission Electron Microscopy. *IEEE Electron Device Lett.* **31**, 854–856 (2010).
7. Hopkins, P. E., Norris, P. M., Stevens, R. J., Beechem, T. E. & Graham, S. Influence of Interfacial Mixing on Thermal Boundary Conductance Across a Chromium/Silicon Interface. *J. Heat Transf.* **130**, (2008).
8. Fujii, Y. Comparison of Surface Roughness Estimations by X-ray Reflectivity Measurements and TEM observations. *IOP Conf. Ser. Mater. Sci. Eng.* **24**, 012008 (2011).
9. Fujii, Y. Improved formulae for X-ray Reflectivity. *Trans. Mater. Res. Soc. Jpn.* **40**, 369–372 (2015).
10. Fujii, Y. Recent Developments in the X-Ray Reflectivity Analysis for Rough Surfaces and Interfaces of Multilayered Thin Film Materials. *J. Mater.* **2013**, e678361 (2013).
11. Esashi, Y. *et al.* Influence of surface and interface roughness on X-ray and extreme ultraviolet reflectance: A comparative numerical study. *OSA Contin.* **4**, 1497 (2021).
12. Hellman, O. C., Herbots, N. & Eng, D. C. A Model for Interdiffusion at Metal Semiconductor Interfaces: Conditions for Spiking. *MRS Online Proc. Libr.* **148**, 83–88 (1989).
13. Yamada, I., Inokawa, H. & Takagi, T. Epitaxial growth of Al on Si(111) and Si(100) by ionized-cluster beam. *J. Appl. Phys.* **56**, 2746–2750 (1984).
14. Majumdar, A. & Reddy, P. Role of electron–phonon coupling in thermal conductance of metal–nonmetal interfaces. *Appl. Phys. Lett.* **84**, 4768–4770 (2004).
15. Fair, R. B. & Weber, G. R. Relationship between resistivity and total arsenic concentration in heavily doped *n* - and *p* -type silicon. *J. Appl. Phys.* **44**, 280–282 (1973).
16. Wang, X., Ho, V., Segalman, R. A. & Cahill, D. G. Thermal Conductivity of High-Modulus Polymer

Fibers. *Macromolecules* **46**, 4937–4943 (2013).

17. Polanco, C. A., Saltonstall, C. B., Norris, P. M., Hopkins, P. E. & Ghosh, A. W. Impedance Matching of Atomic Thermal Interfaces Using Primitive Block Decomposition. *Nanoscale Microscale Thermophys. Eng.* **17**, 263–279 (2013).

18. Bodapati, A., Schelling, P. K., Phillpot, S. R. & Keblinski, P. Vibrations and thermal transport in nanocrystalline silicon. *Phys. Rev. B* **74**, 245207 (2006).
