# Supplementary material for: Inelastic phonon transport across atomically sharp metal/semiconductor interfaces
Source: Nat Commun. 2022 Aug 20;13:4901. doi: 10.1038/s41467-022-32600-w (PMC9392776; doi:10.1038/s41467-022-32600-w)
Supplement: Supplementary file 2 — Lasing Reporting Summary [file 41467_2022_32600_MOESM2_ESM.pdf]

natureresearch

Corresponding author(s): Bo Sun

Lasing Reporting Summary

Nature Research wishes to improve the reproducibility of the work that we publish. This form is intended for publication with all accepted papers reporting claims of lasing and provides structure for consistency and transparency in reporting. Some list items might not apply to an individual manuscript, but all fields must be completed for clarity.

For further information on Nature Research policies, including our data availability policy, see Authors & Referees.

ü Experimental design

Please check: are the following details reported in the manuscript?

1. Threshold

Plots of device output power versus pump power over a wide range of values indicating a clear threshold

- [ ] Yes
- [x] No

The laser power is not crucial for our experiment and we conduct the experiment under a stable power range.

2. Linewidth narrowing

Plots of spectral power density for the emission at pump powers below, around, and above the lasing threshold, indicating a clear linewidth narrowing at threshold

- [ ] Yes
- [x] No

We conduct the experiment under a stable power range, so the linewidth narrowing would not affect our experiment.

Resolution of the spectrometer used to make spectral measurements

- [ ] Yes
- [x] No

We did not use the spectrometer in our measurement.

3. Coherent emission

Measurements of the coherence and/or polarization of the emission

- [ ] Yes
- [x] No

The coherence and polarization of the emission is insensitive to our TDTR measurement, thus we did not measure it.

4. Beam spatial profile

Image and/or measurement of the spatial shape and profile of the emission, showing a well-defined beam above threshold

- [x] Yes
- [ ] No

In Supplementary Information Figure S4

5. Operating conditions

Description of the laser and pumping conditions
Continuous-wave, pulsed, temperature of operation

- [x] Yes
- [ ] No

In Supplementary Information VI, we described the wavelength and the power of the laser we used.

Threshold values provided as density values (e.g. W cm^{-2} or J cm^{-2}) taking into account the area of the device

- [ ] Yes
- [x] No

The density values are not important in our experiment, so we did not measure them.

6. Alternative explanations

Reasoning as to why alternative explanations have been ruled out as responsible for the emission characteristics
e.g. amplified spontaneous, directional scattering; modification of fluorescence spectrum by the cavity

- [ ] Yes
- [x] No

This information is not necessary and sensitive to our experiment.

7. Theoretical analysis

Theoretical analysis that ensures that the experimental values measured are realistic and reasonable
e.g. laser threshold, linewidth, cavity gain-loss, efficiency

- [x] Yes
- [ ] No

In Supplementary Information I, we analyzed the intensity distribution of the laser beam. In Supplementary Information VI, we measured the laser power and ensure the steady state temperature rise is within 10 K.

8. Statistics

Number of devices fabricated and tested

- [x] Yes
- [ ] No

In Supplementary Information Figure S4, we tested the beam spatial shape and intensity profile of our laser. We only use one laser device to conduct the experiment.

Statistical analysis of the device performance and lifetime (time to failure)

- [x] Yes
- [ ] No

In Supplementary Information VI, we used a power meter to measure the laser power, and to confirm the power stability of the laser. We did not analyze the lifetime of the device.
